# Overview and Update on Extracellular Vesicles: Considerations on Exosomes and Their Application in Modern Medicine

**DOI:** 10.3390/biology11060804

**Published:** 2022-05-24

**Authors:** Maria Antonietta Di Bella

**Affiliations:** Department of Biomedicine, Neurosciences and Advanced Diagnostics (Bi.N.D.), University of Palermo, I-90133 Palermo, Italy; m.antonietta.dibella@unipa.it; Tel.: +39-09123865730

**Keywords:** extracellular vesicles, exosomes characterization, drug delivery systems, nanomedicine

## Abstract

**Simple Summary:**

Exosomes are a subpopulation of extracellular vesicles, nanosized particles, lipid bilayer-enclosed, naturally secreted from cells after the fusion of intracellular Multivesicular bodies with the plasma membrane. Their components are proteins, nucleic acids, lipids, and metabolites by which they can act as mediators of cell-to-cell communication. They play a key role in regulating pathophysiological processes, such as immune response, neuronal communication, cancer biogenesis, and regulation. Therefore, they have been largely exploited for their potential therapeutic and diagnostic application. Due to their endogeneity, exosomes present superior biocompatibility and stability in comparison to synthetic carriers, and then they can be used as delivery vehicles. Herein, reviewing recent studies, information on exosome biogenesis, structural characteristics, isolation, and detection current methods is summarized. Further, the obstacles to overcome in the application of exosomes as delivery systems are also considered.

**Abstract:**

In recent years, there has been a rapid growth in the knowledge of cell-secreted extracellular vesicle functions. They are membrane enclosed and loaded with proteins, nucleic acids, lipids, and other biomolecules. After being released into the extracellular environment, some of these vesicles are delivered to recipient cells; consequently, the target cell may undergo physiological or pathological changes. Thus, extracellular vesicles as biological nano-carriers, have a pivotal role in facilitating long-distance intercellular communication. Understanding the mechanisms that mediate this communication process is important not only for basic science but also in medicine. Indeed, extracellular vesicles are currently seen with immense interest in nanomedicine and precision medicine for their potential use in diagnostic, prognostic, and therapeutic applications. This paper aims to summarize the latest advances in the study of the smallest subtype among extracellular vesicles, the exosomes. The article is divided into several sections, focusing on exosomes’ nature, characteristics, and commonly used strategies and methodologies for their separation, characterization, and visualization. By searching an extended portion of the relevant literature, this work aims to give a quick outline of advances in exosomes’ extensive nanomedical applications. Moreover, considerations that require further investigations before translating them to clinical applications are summarized.

## 1. Introduction

Nanomedicine is a term that was used for the first time in 1991 and is defined as the application of nanotechnologies to medicine for the diagnosis, monitoring, prevention, and treating of many human diseases [1].

During recent decades, the applications of nanotechnologies in many biomedical areas have been intensively researched offering excellent results; bio-nanotechnology is an expanding field that requires a multidisciplinary approach including physics, chemistry, material sciences, engineering, informatics, and life sciences. Indeed, nanostructures used for biomarker detection, nano-biochips, nano-electrodes, nano-biosensors, nanostructures for regenerative medicine, nanoparticles (NPs) with antibacterial activities, and NPs for diagnostic applications are currently used [2].

The intrinsic, unique, physicochemical, and mechanical characteristics of particles at the nanoscale level enable their application in theranostics, a new area that combines diagnostics and therapy. Nano-systems can work as image agents or as drug delivery systems because they present an increase in the selectivity and efficacy, and, in comparison to traditional therapeutic molecules, by reducing their accumulation in healthy tissues, they show a decrease in the side effects’ incidence and intensity. In cancer therapeutics, they can offer new solutions to overcome the limitations derived from chemotherapy or radiotherapy [3,4,5].

There are different types of synthetic NPs made from various materials including, for example, polymerNPs, solid lipidNPs, crystalNPs, liposomes, micelles, hydrogels, and dendrimers. Nevertheless, each of these formulations, organic or inorganic, presents its advantages and disadvantages for nano-diagnostic or nano-therapeutic applications [6,7].

The artificial or engineered NPs obtained by chemical synthesis pose several limiting factors for their application in vivo, such as:

(a) Their constituents must be evaluated for potential in vivo toxicity (genotoxicity, immunotoxicity, cellular stress, inflammation) before clinical application; currently, several aspects of the interactions of NPs with the biological systems have not been understood completely.

(b) Large-scale production could be limited. Nanomedicine manufacturing processes include multiple steps that can be easily controlled on a small scale. However, achieving the optimization of the formulation at a large scale is still challenging.

To overcome these limitations, many research groups have focused their attention on natural NPs as a useful tool for nanomedicine, given their good qualities [3].

The information included in this review article with emphasis on recent publications aims to briefly summarize the potential use of some natural nano-systems and the most relevant approaches for the application as therapeutic agents and drug delivery carriers. The source for the retrieval of the literature contained in this work was the PubMed database.

## 2. Natural Nanoparticles Versus Synthetic Nanoparticles: The EVs Benefits

Among naturally occurring NPs, extracellular vesicles (EVs) are heterogeneous nano-sized structures released constitutively into the extracellular space by almost all cell types in both prokaryotes and eukaryotes [8,9,10,11].

EVs have an interior aqueous core surrounded by a lipid bilayer membrane and contain several components to be delivered to surrounding or distant target cells. Growing evidence poses attention on the EVs and their pivotal role as vehicles for cell-to-cell exchanges. As a matter of fact, they can influence the microenvironment via the direct and protected transfer of bioactive molecules, receptors, and effector molecules with effects that can be protective and pathologic [12,13,14].

In humans, EVs have been detected in different tissues including tumors, blood, and in several body fluids such as breast milk, saliva, urine, ejaculate, and cerebral spinal fluid [15,16,17,18,19,20,21].

Owing to their natural origin, EVs present good biocompatibility and low toxicity; they present enhanced stability in the blood due to the evasion of the immune system. In addition, they have limited immunogenicity and are biodegradable. These properties make them advantageous over synthetic vehicles or NPs [22,23,24,25]. Another advantage of EVs is their target potential; they can cross physiological barriers such as the blood–brain barrier (BBB) so that they may be home to a specific neuron population. Moreover, the elements naturally present in them can also act in collaboration with encapsulated drugs, creating thus a synergic effect [26,27].

## 3. EVs Population under Microscope

**Figure 1:**
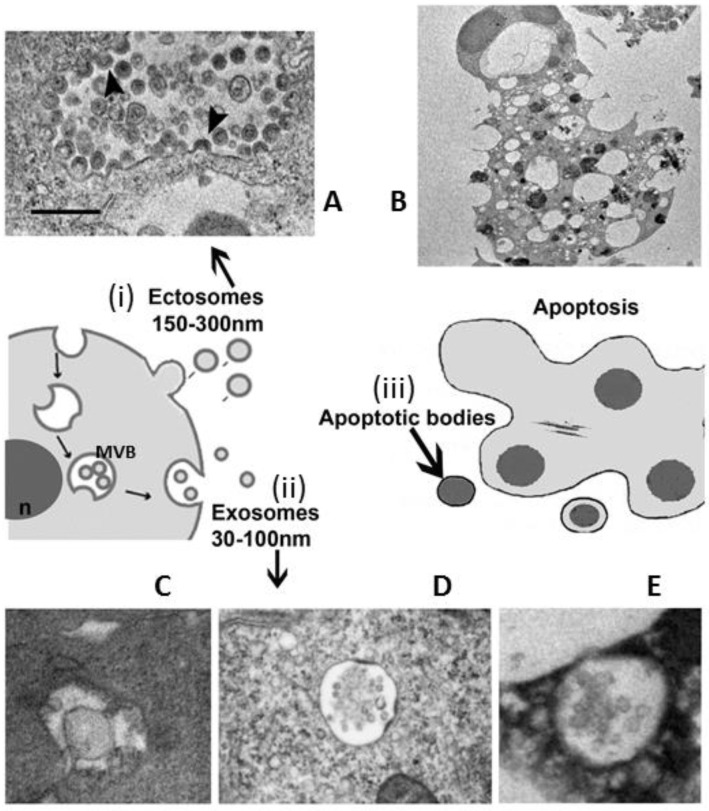
Deployment of extracellular vesicles to exchange biological molecules contained within a membrane boundary. (i) Budding of microvesicles off the plasma membrane (ectosomes or microvesicles); (ii) release of exosomes through the fusion of multivesicular bodies (MVBs) with the plasma membrane; (iii) blebbing off of larger vesicles, especially from apoptotic cells (apoptotic bodies). In the upper side there are representative transmission electron images: (**A**) microvesicles liberation (arrowheads), and (**B**) an apoptotic cell. In the bottom side: (**C**) inward invaginations of the MVB indicate the beginning of exosomes biogenesis, (**D**) MVB enclosing a lot of exosomes, and (**E**) MVB near to fuse with the membrane. n = nucleus.

Traditionally, EVs have been classified into three major types, depending on their size and biogenesis prior to secretion:

- apoptotic bodies (ApoBDs): vesicles of relatively large size (1–5 μm), variable in structure and composition; they are released by the blebbing process of cells undergoing apoptosis (Figure 1B)
- microvesicles (MVs): diameter size of 150 nm–1 μm, they are shed directly by the outward budding and fission of the plasma membrane (Figure 1A)
- exosomes (EXOs): EVs with a diameter size ranging from 30 to 150 nm, with a density of 1.13 to 1.19 g/mL in sucrose; they originate from the late endosomal trafficking machinery. They are intracellularly produced into organelles called multivesicular bodies (MVBs) and ultimately, they are released into the extracellular milieu as a result of MVBs fusion with the plasma membrane (Figure 1C–E) [9,10,11,28,29].

Currently, several methods are used to characterize isolated EVs. Conventional Western blotting, mass spectrometry, flow cytometry (bead coupled), microfluidics chips, polymerase chain reaction, and nuclear magnetic resonance (NMR) have been utilized to identify protein composition, to detect nucleic acids and lipids. Traditional and emerging technologies sometimes are not sufficient to characterize vesicles and have complete qualitative and quantitative information. Thus, EVs need often to be visualized with super-resolving techniques. Presently, electron microscopy (EM) is the only method that allows determining simultaneously their size, shape, morphology-integrity, inter-particle interaction, and spatial relationship with tissues and cells [30,31].

Although emerging detection methods and new analytical techniques are often used for comprehensive characterization, the analysis of EVs remains as no available purification method can strictly separate them based on their size. Moreover, there is currently no consensus on specific markers that uniquely distinguish the origin of these vesicles once they have left the cell. In particular, the possibility to discriminate EXOs from MVs is still largely debated [32]. Recently, guidelines for working with EVs were suggested in MISEV2018. The International Society for Extracellular Vesicles (ISEV) board members recommended using the term extracellular vesicles for “*all particles naturally released from the cell that are delimited by a lipid bilayer and cannot replicate*”; they suggested combining EV extraction methods and improving techniques for purification characterization in order to validate and replicate the results of experiments. The scientific community is currently oriented “*to consider use of operational terms for EV subtypes*” distinguishing them on “*physical characteristics such as size, small EVs (sEVs)* and *medium/large EVs (m/lEVs), with ranges defined, for instance, respectively, < 100 nm or < 200 nm [small], or > 200 nm [large and/or medium])*” [33].

## 4. The Use of Exosomes for Clinic Aims

Among the large subpopulation of EVs, ApoBDs are quite variable in size and content; they are typical membrane blebs released by cellular disassembly that are quickly phagocytized by surrounding cells. The apoptotic bodies can harbor different biomolecules: genomic DNA, histones, fragments of the cytoplasm, and intact organelles Their formation represents the expression of both cell clearance and intercellular communication, but little is known about their function compared to the progress in MVs and EXOs investigation [34,35,36,37]. Indeed, in the past decade, the scientific interest in MVs and EXOs has rapidly increased; their functional characteristics and the role played by them in human physiology and in the pathogenesis of major human diseases have received extensive attention.

MVs are heterogeneous structures classified as large EVs, incorporating nucleic acids, lipids, and many proteins [38,39].

Depending on the specific purpose, both MVs and EXOs have merits as potential therapeutic systems.

The following section will focus only on the biological importance of the small vesicles called EXOs and will summarize the promising approach based on their attractive properties such as their ultra-small dimension, spherical shape, and molecular components. As a subtype of EVs, EXOs show circulation stability (in particular, some of them are positive for the CD47 transmembrane protein, a signal that protects cells from mononuclear phagocyte systems and increases the time of their circulation) [40]. They show the ability to bypass biological barriers and accumulate to pathological sites; EXOs are easily excreted out of the body. Hence, they could find application in the diagnosis, prevention, and treatment of diseases [41].

### 4.1. EXOs Characteristics

For many years, researchers have considered EXOs as waste products obtained from the shedding of plasma membranes. After decades, this image of waste bins changed to that of biologically active particles. Later, the term “exosome” was coined [42,43,44,45,46,47]. Since these initial discoveries, the field of EXOs research has enriched considerably as confirmed by the number of publications over the years, and dramatically increased since 2010 by about 50 times according to PubMed. 

EXOs are characterized by various types of complex cellular components present both on the surface and packaged inside the lumen after selective mechanisms. To date, omics approaches (proteome analysis, lipidomics, metabolomic analysis, etc.) have identified numerous proteins, nucleic acids, lipids, and metabolites.

Proteins include both membrane and cytosolic components: some surface receptors, adhesion proteins, integrins, cytoskeletal proteins, membrane transport, and fusion-related proteins. The presence of TSG101 and other accessory proteins that are involved in the formation of intraluminal vesicles (ILVs) denotes that the EXOs’ biogenesis pathway depends on the endosomal sorting complexes required for transport (ESCRT). Tetraspanin proteins and phospholipids that play a critical role in membrane biogenesis denote an ESCRT-independent alternative mechanism of biogenesis. Tetraspanins, heat shock proteins (Hsp), and other related proteins involved in MVB production are the most conserved proteins [48,49,50].

Some proteins are specifically related to the nature of the cell and tissue of origin. Different EXOs have specific proteins that reproduce the status of the parental cell. That is one of the reasons for their heterogeneity. For example, T-lymphocyte-derived EXOs have enzymes and perforin on their surface. Antigen-presenting cells (B lymphocytes, dendritic cells) (APCs)-derived EXOs contain major histocompatibility antigen complex (MHC, MHC-I, MHC-II). Tumor-specific proteins are contained in many EXOs from tumor cells [51].

**Figure 2:**
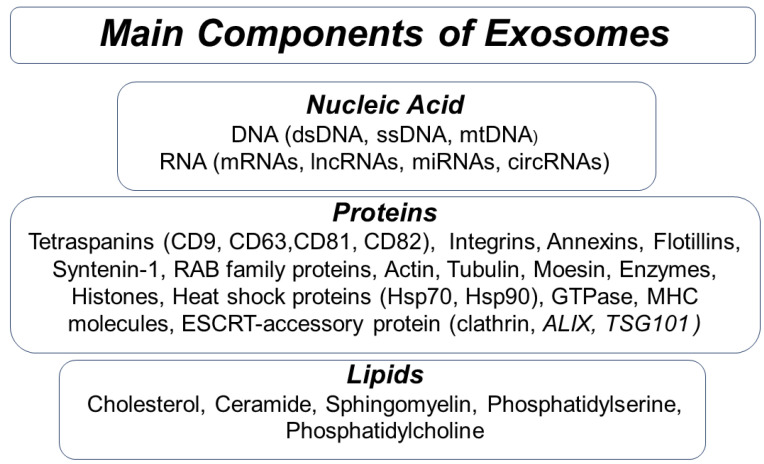
Molecular composition of exosomes. These vesicles contain different cell surface proteins, membrane transport and fusion, antigen presentation, signal transduction, targeting, and adhesion proteins. Several molecules of nucleic acids and lipids are present.

EXO cargos also consist of nucleic acids, such as genomic DNA, that can induce phenotype switch, messenger RNA (mRNA) that can be translated in target cells, microRNA that can mediate RNA silencing, circular RNA, long non-coding RNA, and viral RNA. Lipid rafts are present on the surface of EXOs (Figure 2). Based on the specialized EXOCARTA database (http://www.exocarta.org accessed on 28 April 2022), exosomes contain 9769 proteins, 3408 mRNAs, 2838 miRNAs, and 1116 lipids; (see references [51,52]).

Upon their release in the extracellular space, EXOs can be destroyed, or migrate to interact with other cells. The recipient cells may be in proximity or be present at a distant site; the vesicles can travel over large distances being carried via the blood or lymphatic circulation. The lipidic membrane of EXOs acts as a protection barrier to cargo and the signal arrives undiluted and protected from damage bypassing phagocytosis. Molecules such as nucleic acids that could be degraded in the extracellular milieu are protected from enzymatic degradation (for example, RNAases) [13].

**Figure 3:**
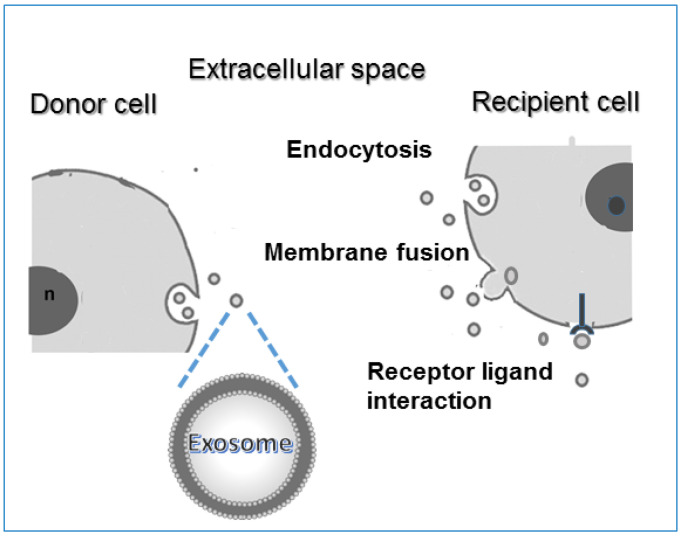
Schematic representation of the exosome uptake: exosomes released into the extracellular space from donor cell enter target cells in three ways, which are direct fusion with the plasma membrane, endocytosis, and protein–receptor interactions.

The major ways of internalization of EXOs entering the recipient cell are the direct fusion to target cell plasma membrane, endocytosis uptake, or receptor–ligand interactions [53,54,55] (Figure 3).

When EXOs enter target cells, their cargo can be accepted by recipient cells. Once released inside the target cell, the exosome cargo can perform a variety of functions that, depending on the cellular origin, regulate a plethora of physiological activities. EXOs may affect gene expression, regulate metabolism, participate in responses during microbial infection, and facilitate disease progression. Over the past decades, most of the research attention in this field has been focused on exploring the vital roles as long-range messengers of these vesicles [56,57,58,59,60,61,62].

The amount of EXO biogenesis in different cells depends on their physiological and/or pathological states. Cellular stress and signals activation can modulate their excretion. Accumulating evidence shows that cancer cells release a larger amount of EXOs compared to normal cells; tumor-derived EXOs can contribute to cancer growth by inducing anti-apoptotic and oncogenic pathways such as invasion, metastasis, and angiogenesis. Some EXOs can promote tumor immune evasion with T-cell apoptosis induction. They are also responsible for epithelial–mesenchymal transition and interconversion to mesenchymal–epithelial transition in several human malignancies. For the above reasons, it is important to take into consideration the function of secreting cells to ensure the safety of these nanocarriers in clinical applications [63,64,65,66,67].

### 4.2. EXOs Source

As mentioned before for EVs, EXOs in vivo are released in many biological fluids (such as synovial fluid, breast milk, urine, and saliva), amniotic liquid, blood serum, and malignant effusions of ascites among others. Neuronal cells, fibroblast cells, adipocytes, intestinal epithelial cells, and tumor cell lines produce EXOs in vitro.

Different studies have suggested that several types of cells seem to be more eligible to produce EXOs for therapeutic purposes and that not all cell-derived vesicles are ideal as drug carriers. Drug capacity and efficient delivery depend on the size, yield, intracavitary composition, and surface proteins that mirror the cell and tissue of origin [68].

The major types of used EXOs are derived from:

- *Dendritic cells* have been used due to their low immunogenicity. Interestingly, their EXOs still maintain this immune function. Dendritic cells-derived EXOs can overcome the biological barriers, such as the blood–brain barrier [69,70,71,72].
- *Macrophages* are mononuclear phagocytes that have critical roles in innate immunity. Macrophage-derived EXOs are known to express functional immune proteins; they can interact with brain vessel endothelial cells and cross the blood–brain barrier, an ability mediated in part by surface components; they can deliver some factors such as anti-inflammatory cytokines (i.e., IL-4). Moreover, they exhibit strong anti-tumor and anti-inflammatory effects [73,74].
- *Mesenchymal stem cells* are a popular choice for cell therapy. Indeed, they are easily obtained from different human tissues such as bone marrow, dental pulp, and adipose tissue. Mesenchymal stem cells are capable of self-renewal and are involved in modulating the immune response. EXOs isolated from these cells are extremely beneficial in promoting wound healing and in repairing tissue such as skin and cardiac tissue. Cao et al. [75] found that mesenchymal stem cell-derived exosomal miR-125b-5p could promote the repair of renal tubules in acute kidney injury. These vesicles also seem to inhibit cancer progression and have an inflammation melioration capacity. Additionally, these cells are known to secrete relatively high numbers of EXOs [76,77,78,79].
- *Cancer cell lines* such as melanoma cells are commonly used to produce EXOs. As reported before, tumor cell-derived EXOs can either block tumor growth or be involved in cancer progression and are capable of converting a normal cell into a transformed one. Thus, more importantly, tumor cells may be a double-edged sword when used for delivering therapeutics agents because their EXOs could show potential risk in aggravating a patient’s malignity instead of improving it or conferring drug resistance [80,81].
- To overcome the risk of horizontal gene transfer when EXOs are recovered from tumor cells or immortalized cells, some researchers have investigated the potential of human *Red Blood Cells* (RBC) as a source of vesicles. RBCs are abundant in the body, easy to obtain, and available in blood banks. A strategy to generate large-scale amounts of RBC-EXOs for the delivery of RNA and drugs was demonstrated by Usman et al. [82].
- Plasma exosomes are also derived from *Platelets* (PLT). These originate from bone marrow megakaryocytes and have no nucleus and a short half-life. PLT-derived EXOs can be obtained from animals, healthy volunteers, and from platelets in disease states. The functions of PLT-EXOs depend mainly on their source as they are rich in a variety of cargos. Platelets in disease states often contain pathogenic factors that can be used as biomarkers for disease diagnosis. EXOs obtained from healthy volunteers or mice can inhibit platelet activation and endothelial inflammation, while human PLT-EXOs have been shown to increase cell proliferation and migration of mesenchymal stromal cells (MSCs) from human bone marrow. PLT-EXOs could present advantageous therapeutic properties, including homologous administration in the clinical setting, thus overcoming the restrictive requirement of other biological products. Although procedures such as high-speed centrifugation of plasma induce the aggregation of PLT-derived EVs more than erythrocytes EVs and washing for preparing ‘washed’ platelets shows that most EXOs will be removed, nowadays isolation protocols with the use of specific commercial kits can avoid this effect [19].

A summary of cell lines used to produce EXOs for clinical applications can be found in the updated review [68,83].

Recently, vesicles obtained from plant cells or food have gained attention from researchers. Bovine milk EXOs have been studied as a viable alternative of high impact in drug carriers due to their lack of toxicity, on account of their biocompatibility, and stability in an acidic environment so that they can be delivered orally [84,85].

Exosome-like nanoparticles (ELNs) derived from tissues, organs, apoplastic fluid, and the juice of several plant species such as ginger, lemon, grapefruits, and carrots are characterized by various good properties that make them suitable for clinical applications. ELNs seem to contribute to the plant defense in response to pathogens, and they are also known for their anti-inflammatory and tumor growth-suppression properties. Moreover, they seem to localize in tissue and remain intact inside cells after administration, confirming the possibility that they can be used for intracellular drug delivery [86,87,88]. As regards their composition, they show a lipid bilayer structure comparable to that of mammalian cell-derived EXOs and artificially synthesized liposomes; contrary to those, they lack cholesterol, present a higher percent of phosphatidic acids (a cell-signaling component with many biological activities), phosphatidylcholine, and phosphatidylethanolamine [89]. A variable number of proteins and miRNAs have been identified in plant-derived EXOs [88,90].

However, although the morphology and composition of these ELNs are like mammalian exosomes, despite the several advantages of food and vegetable-derived vesicles, and despite the possibility of their production in large quantities from fresh plant juice, further studies are still required. Information on their biogenesis is still lacking and it is an urgent task to characterize and clearly identify markers of plant EVs. At present, research on the biogenesis of plant EVs is not sufficient and needs to understand all details of their mechanism of action before they can largely be introduced as nano-platforms in medical practice.

### 4.3. EXOs Isolation and Storage

Multiple methods and different ways have been established to isolate plant and animal EXOs from biological fluids or in vitro cell culture supernatant. For different purposes and applications, it is very important to select the producer cells, and it is essential to understand the characteristics of the different isolation approaches that enable large-scale bio-manufacturing and assure the efficacy of the strategy. Indeed, it is known that certain procedures may compromise the EVs’ integrity and structure or can be associated with aggregate formation and cargo impurity [91].

**Figure 4:**
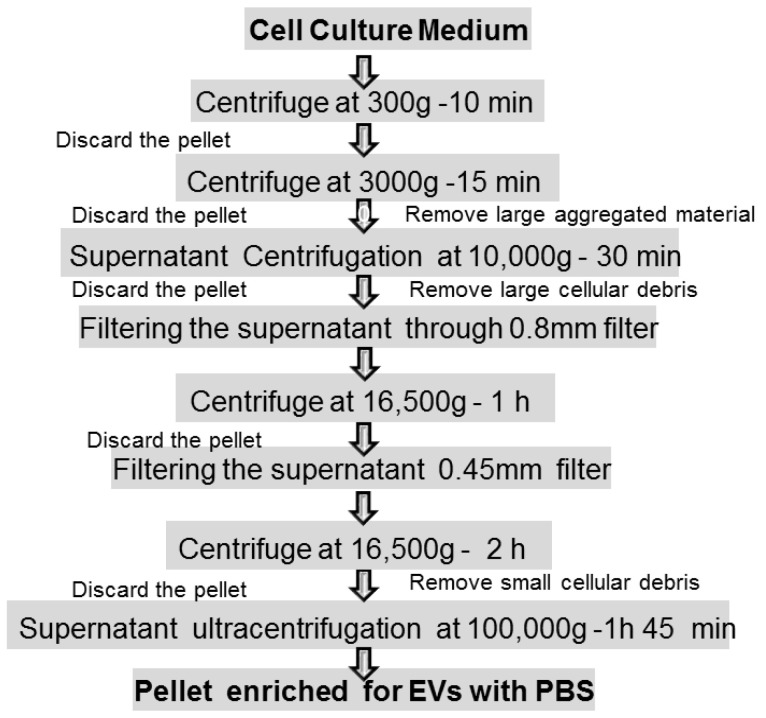
Steps of differential ultracentrifugation for exosome isolation from cell culture medium.

Among the different isolation methods, the most traditional and commonly used are:

- *Ultrafiltration:* is a method based on the vesicle size, involving the use of fluid pressure to drive the migration through a polymeric membrane with defined pore size; vesicles are separated selectively from the samples with the simultaneous retention of larger molecules. It is simple and fast, but EXOs can be degraded and lost [92].
- *Immunoaffinity:* is a capture isolation technique based on the recognition by antibodies or ligands of EXO marker components (antigen) that are exposed on the vesicle surface. The immunoaffinity method has the advantages of rapid isolation, simplicity, and high specificity, and the sample volume can be very small in comparison to ultracentrifugation, but it is very expensive due to the cost of antibodies [93].
- *Size-Exclusion Chromatography (SEC)* techniques can isolate EXOs based upon molecular size and density, mainly by means of a column filled with a porous stationary phase with a specific pore size distribution. When the sample enters the gel, small particles with small hydrodynamic radii diffuse into the pores while large molecules with large hydrodynamic radii will not. Hence, the passage of proteins and other smaller contaminating molecules is delayed while larger molecules or larger vesicles (>75 nm) exit the column and will be eluted earlier in the void volume The porous stationary phase contained in the column can be cross-linked dextrans, polyacrylamide, agarose beads (commercially named as Sepharose), and allyldextran in which small particles can penetrate. The primary advantages of this technique are the screening of high-purity EXOs with less protein contamination compared to ultracentrifugation, and the preservation of vesicle integrity, structure, and biological activity as it relies on gravity rather than sheer force for isolation. However, this technique is limited by: (1) the need for dedicated equipment; (2) the accessibility of the chromatography column to contamination; therefore, aseptic working conditions should be ensured especially if the isolated EVs are intended for therapeutic use; (3) an initial large volume is required; (4) low yield; (5) difficulty in scaling up; (6) inability to separate EXOs from vesicles of the same size. Research efforts have been performed to overcome those challenges and enhance SEC efficacy and speed. For instance, the EXO pellet is re-suspended after enrichment by ultracentrifugation in combination with ultrafiltration methods and then further purified using SEC. This combined strategy resulted in improved purity and preserved EXO function. Moreover, commercially available columns and kits based on size-exclusion chromatography were designed to simplify EV isolation; iZON Science produced the qEV Exosome Isolation Kit that, as well as the PURE-EVs kit (Hansa Biomed), allows rapid, high-precision isolation within less than half an hour so the SEC methodology is nowadays relatively easy and fast. However, this combination is not suitable for scale-up production [94].
- *Microfluidics* platforms represent emerging isolation methods developed to separate EVs from large cellular debris and protein aggregates. Microfluidics techniques enable the differentiation, capture, enrichment, and isolation of particles of very similar shapes and sizes. Different isolation principles have been designed: size based, immune-affinity based, and dynamic categories that make use of emerging nanomaterials. Size-based exosome separation devices allow the separation of highly pure EXOs driving the plasma inside a channel where nanofilters, nanoporous membranes, or nanoarrays can trap vesicles when fluids flow through them. In another device, an acoustofluidics device, using ultrasound standing waves, in a contact-free continuous flow manner, EXOs are directly isolated from undiluted small blood samples based on their size, density, and compressibility. The result is the formation of clusters of EVs. These clusters are then washed and released upon deactivation of an ultrasound. This device maintains the structures, characteristics, and functions of the EXOs with a purity of about 98%. In addition, it enables the separation time, reagent consumption, and sample volume for isolating EXOs to be significantly reduced with short processing times with decreasing human intervention. In an immunoaffinity-based microfluidic device, the vesicle separation relies on specific biomarkers on the EXOs membrane. A commercial immune-microfluidics chip (ExoChip) allows the isolation of EXOs from mixed cultures because it is functionalized with a commonly expressed antigen, CD63. The specific interactions between CD63 and antibodies immobilized on the chip allow the capture of the vesicles. Unfortunately, to separate them efficiently, the immunoaffinity-based separation microfluidic devices need highly represented antigens (targeted proteins) on the vesicle surface. Other innovative and attractive separation approaches that have the ability to isolate EXOs based on their physical and biochemical properties are being simultaneously developed: some microfluidic isolation methods typically require small starting volumes from serum and cell culture (10 s–100 s of μL), while others can be performed on larger volume samples; they can reduce reagent consumption, are fast, and efficient. However, scalability, validation, sample pretreatments, and standardization are still considered bottlenecks for these devices, which are mainly applied in the field of diagnosis [95,96].
- *Ultracentrifugation* (100,000× *g* or greater) is currently the most widely used purification method that mainly depends on vesicle density, size, and shape. It consists of two steps after pelleting down cells: a pre-cleaning and filtering of samples centrifuged at low and intermediate speed centrifugation (500–10,000× *g*) to remove dead cells and cell debris, followed by the flotation in a density gradient centrifugation to precipitate and enrich EXOs. High-speed centrifugation (40,000–100,000× *g*) is often combined with a density gradient using commonly iodixanol or sucrose as a medium to remove contaminants such as proteins, protein/RNA aggregates, and lipoproteins. The EXOs can be collected in the density range of 1.1 to 1.2 g/mL Depending on the rotor utilized, this procedure is suitable for large sample processing; it requires little sample pretreatment and has the characteristics of low contamination risk. Moreover, the affordability is high since only one ultracentrifuge is needed for long time use. Apart from the access to expensive equipment, it is of low cost. At the same time, however, the density gradient centrifugation method is time consuming and requires extra care to prevent gradient damage. In addition, damage to EXOs by high-speed centrifugation might occur if used for long times (more than 3–4 h) [97] (Figure 4).
- *Co-precipitation* is an appealing precipitation-based isolation method thanks to the simple protocol and high yield. Polyethylene glycol (PEG) is generally used as a co-precipitator by decreasing the solubility of EXOs. The method lacks specificity and results in low purity of vesicles [98].

Even if various and easy-to-use commercial kits are now available on market, to date, there is still no ideal “gold” EXO isolation method; the low purity and high cost of the preparations restrict their utility. Sometimes the combination of different isolation methods may be better in accordance with the purity required and the sample volume [99].

Nevertheless, the storage conditions and preservation of produced EXOs that must be used for therapeutic applications need to be fully elucidated as they can affect the amount and the quality of the final product. Several data suggest that the stability of vesicles from different origins may also be different. To date, storage at −80 °C in phosphate saline buffer is the most used way to preserve EXOs [100].

### 4.4. EXOs Clinical Applications

**Figure 5:**
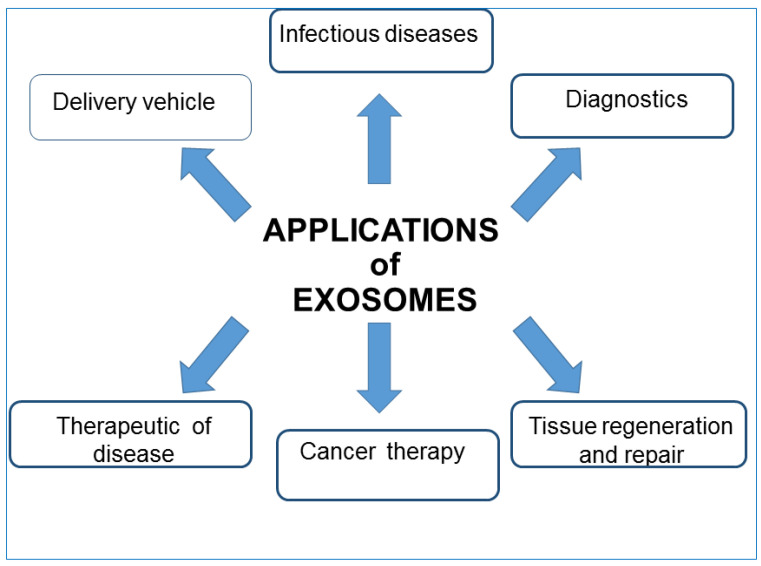
Some of the major uses of exosomes in clinical development. Exosomes affect various aspects of cell biology and can find employment in different fields of nanomedicine.

The clinical applications of EXOs can be summarized as follows:

- *Liquid biopsies*: because EXOs differ in their composition based on the current state of the secreting cells, being able to isolate them from different body fluids can be considered a potent screening tool. Compared with traditional solid biopsy, liquid biopsy has a number of advantages: firstly, minimal trauma. Thus, EXOs isolated from liquid biopsies can be used as both diagnostic and prognostic non-invasive biomarkers. EXOs released from normal and cancer cell lines have different nucleic acid contents and membrane structures in accordance with their surface proteins, cholesterol contents, and cholesterol/phospholipid ratios. This enables the early detection of many pathological conditions, and their regression or progression in response to therapy. EXOs originating from tumor cells possess active molecules and specific genomic and proteomic features characteristic of a particular tumor type; therefore, their analysis could predict the potential presence of the tumor. For example, human serum exosomal long noncoding RNAs-UCA1 and exosomal miRNAs can be used as diagnostic biomarkers for cancer risk [101,102,103]. Epidermal growth factor receptor (EGFR), placental alkaline phosphatase (PLAP), and leucine-rich alpha-2 glycoproteins (LRG1) are potential biomarkers for non-small cell lung cancer, as they are all overexpressed in patients. Moreover, Grimolizzi et al. found that in both early and advanced-stage non-small cell lung cancer patients, miR-126 was mainly present in EXOs, while in healthy controls, circulating miR-126 was equally distributed between EXOs and exosome-free serum fractions. The detection of prostate cancer can also be achieved, evidencing the presence of exosomal miRNA-141 and miRNA-375 [104,105,106]. EXOs can find application as biomarkers also in cardiovascular diseases, and exosomal miRNAs may be beneficial for diagnosing heart diseases. Another important disease that could benefit from the study and application of EXOs is diabetes mellitus. Recent literature demonstrates that the content of exosomal miRNA is remarkably different in the sera of type I diabetes patients in comparison with that of healthy control. In addition, a pre-clinical study has indicated that exosomes also participate in type 2 diabetes pathogenesis. Certain EXOs biomarkers (P-S396-tau, P-T181-tau, and Ab1–42) seem to predict the development of Alzheimer’s disease up to 10 years in advance; EXOs secreted by various parts of the kidney, contain several biomolecules that might be markers of abnormality present in the kidney [107,108].
- *Therapeutic intervention*: Several studies have highlighted the therapeutic importance of EXOs. Being able to redirect vesicles to tissues of interest, EXO administration could be used to degrade pathological signals or focus their intrinsic therapeutic activity. EVs regulate various normal physiological and pathological activities; thus they can be used as natural therapeutic agents for treating a variety of common diseases. There are sufficient pre-clinical studies to support the application of dendritic cell-derived EXOs to treat different types of cancer such as metastatic melanoma and non-small cell lung cancer. For example, EXOs derived from mature dendritic cells prevent the production of cancer cells as they contain DHA (C22:16 docosahexaenoic acid, fatty acid), which enhances the antigen-presenting ability of cells and thus inhibits tumor cell proliferation. However, as EXOs participate in the progression of tumors and promote various stages of tumorigenesis, some research aims to regulate the process of EXOs secretion and reduce their release from tumor cells to normal levels or inhibit their uptake by the target cells [109]. The results of a preclinical trial indicated that by using dimethylamiloride (DMA), the secretion of EXOs can be repressed in murine tumor models by blocking intracellular Ca^2+^ and Na^+^/Ca^2+^ and Na^+^/H^+^ channels. Indeed, the increase in intracellular Ca^2+^ and reduction in intercellular and intracellular pH values lead to an increase in EXOs secretion, and the consequent uptake by recipient cells. Moreover, in order to remove the metastatic effect of cancer, a biotechnology company named Aethlon Medical has developed an adjuvant therapy called HER2O-some, which decreases HER2-positive EXOs secreted by cancer cells in circulation and thus interrupts the progression of HER2-positive breast cancer. Although the technique based on EXOs removal has achieved great progress, further research is still needed to assess its clinical safety. EXOs derived from bone marrow mesenchymal stem cells could produce protective effects in brain injury models, multiple sclerosis, and other neurological disorders thanks to their ability to enter biological barriers such as the BBB. In epilepsy, the administration of native EXOs can result in a reduction in inflammation, memory preservation, and a decrease in neuronal loss. The regenerative properties of EXOs have been shown after stroke injury in both rat and mouse models. By means of proteomics analysis, EXOs derived from mesenchymal stem cells were found to contain various proteins involved in the process of brain repair function. EXOs may accelerate and stimulate regeneration in several tissues, for instance, kidneys, and also seem to modulate transplant rejection [78,110,111]. Furthermore, EXOs have shown a protective effect on joint damage in a collagenase-induced OA model and in several cardiovascular diseases [112,113]. EXOs can act as a decoy for virus and bacterial toxins, thus suggesting a potential role as therapeutic agents [114,115]. These days, well-designed EXOs against COVID-19 may be feasible to prevent initial infection or further internal dissemination of the virus, thus reducing the virus burden and disease severity. Interestingly, EVs can be used in the treatment of COVID-19-associated brain damage due to their unique ability to penetrate the BBB and their potential to be engineered and targeted to a specific part of the CNS [116,117,118,119,120]. Recently, clinical trials that point to the use of EXOs as therapeutic agents against COVID-19 infection are currently ongoing. Moreover, EXOs are also being explored for their vaccine potential. In order to overcome the shortcomings of existing vaccines and contain escalating cases of COVID-19, several biotechnology companies are focusing on vaccine development using EXOs as a platform against SARS-CoV-2 [121]. In the following section, it will be in short reported how the potential use of natural EXOs is largely improved when they are modified and used as carriers of therapeutic agents.
- *Drug delivery and nanotherapy*: any shuttle used for drug delivery must possess several necessary characteristics: (i) can encapsulate an adequate amount of drug to obtain therapeutic effect; (ii) must possess a prolonged inherent stability of size, structure, and bioactivity of the therapeutic agent during circulation before reaching the target organ; (iii) can evade macrophages’ phagocytosis, must have non-toxic properties, be biocompatible with the immune response, and be non-immunogenic. For the past few years, several new nanoscale systems to deliver therapeutic drugs or genes have been designed to improve bioavailability, reduce the toxicity of traditional drugs, and target specific sites. The first clinical success in nanotechnology occurred in 1995 with the approval of Doxil (a formulation of liposomal doxorubicin). Since then, new therapies and biocompatible nanocarriers have been designed (silver nanoparticles, polymer nanoparticles, nanotubes) and used, but until now, an ideal drug delivery system, with long-term safety and biocompatibility, remains to be planned. EXOs are good candidates for delivering vehicles of chemotherapeutics agents to specific cells and tissues and trigger phenotypic changes. EXOs have lower immunogenicity than virus-based delivery systems and liposomes. As aforementioned, the lipid bilayer gives EVs an amphiphilic nature that allows them to store and dissolve both hydrophobic and hydrophilic compounds. Compared with free drugs, exosomes loaded with chemotherapeutic drugs showed a higher efficacy. Examples of such systems are doxorubicin-loaded EXOs, EXO-curcumin, and paclitaxel-loaded EXOs that were shown to exert stronger anti-proliferative activities or cytotoxicity in cancer cells than drugs alone. Curcumin is a polyphenol compound made from turmeric, a flowering plant of the ginger family. Curcumin loaded onto exosomes forms a complex that improves its solubility, stability, and bioavailability enabling to exert its antioxidant, antineoplastic, anti-inflammatory, and chemopreventive properties. Sun et al. treated mice with this complex and found that mice were able to resist lipopolysaccharide-induced septic shock [122]. Paclitaxel is a highly hydrophilic molecule used as an antitumor drug, but its clinical application is limited because of dose-dependent toxic side effects. The toxicity resulted in reduced exosomes loaded with this drug. Yang et al. using the zebrafish model demonstrated that exosomes loaded with the doxorubicin drug were able to cross the BBB and inhibit the growth of tumors. Doxorubicin is an amphiphilic drug that inhibits angiogenesis and controls tumor growth [123]. In addition, molecules such as catalase with antioxidant properties, anthocyanins with anti-cancer activity against ovarian cancer, and other molecules can exert increased therapeutic effects when loaded on exosomes. EXOs can also deliver DNA and RNA as genetic therapeutic agents. These molecules have sizes that obstacles passive diffusion and they are susceptible to enzyme degradation. Thus, they can be delivered and protected by the double membranes of the exosomal carrier. A summary of EXOs clinical applications is reported in Figure 5.

Several methods have been developed to incorporate proteins or nucleic acids into EXOs. Some of them can be performed on donor cells as the composition of the EXOs is highly controlled within cells. Thus, the incorporation of pharmaceutical components can be achieved by:

1. *An active approach*: donor cells are co-incubated with small molecular weight drugs or other chemical compounds. Cargos may passively diffuse across the cell membrane and concentrate in the cytoplasm; after appropriate stimulation such as heat or hypoxia, cells release EXOs loaded with the desired cargo. A simple over-expression in the parental cells of desired cargo is most of the time sufficient. The gene transfection approach is used for loading exogenous nucleic acids into donor cells; the cells are transfected with DNA plasmid vectors, noncoding RNAs, etc., that are easily packaged by the natural biomolecular synthesis processes within EXOs. Then, EXOs can be rapidly isolated and purified [124]. This approach is simple but can result in poor loading and thus is not suitable for wide applications.
2. *A passive approach:* EXOs previously isolated from different sources are incubated with various molecules, preferentially hydrophobic, that can easily penetrate inside and localize in their lumen. To improve EXOs permeabilization, different chemical or physical methods can be used. For example, saponin permeabilization, sonication, and mechanical extrusion over a polycarbonate membrane. A method often used for loading siRNA is electroporation; following the application of high-voltage electricity to the suspension of EXOs and therapeutic agents, temporary pores are created in the membrane through which molecules can pass inside the vesicles [125]. Another approach to modifying vesicles and improving their specific targeting ability is surface membrane modification. Using procedures such as chemical modifications to the EXO membrane, click chemistry, etc., target molecules, peptides, and ligand aptamers are allowed to directly anchor on the exosomal membrane [126,127].

**Figure 6:**
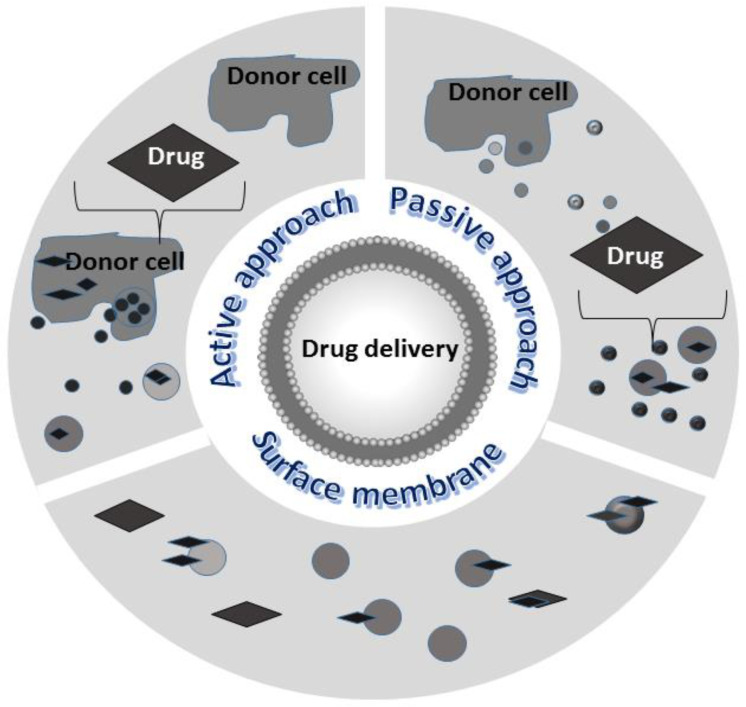
Main strategies for loading exosomes with non-native cargos: cellular modifications or direct vesicle modifications can generate enhanced exosomes to be used as delivery vehicles.

These applications are represented in Figure 6. All these approaches have pros and cons: in the pre-secretory approach, the drug loading efficacy cannot be controlled; in the post-secretory approach, EXOs aggregation or membrane damages can occur; the surface modification can affect the original biological activity of the EXOs.

Once the EXOs with the desired cargo are produced, the liberation of their content to the recipient cells occurs by different mechanisms and consequently, the cellular phenotype can be altered. Different methods of modification and functionalization of EXOs have been designed by researchers, but further insights into these reactions need to be acquired to completely understand these strategies and obtain better loading efficiencies [128,129].

## 5. Exosomes Characterization and Detection Techniques

In order to correlate the EXOs structure and properties, it is necessary to determine their morphological analysis, size distributions, shape, composition, surface charge, and biochemical properties. By definition, the nanovesicles lie below the diffraction-limited resolution of conventional light microscopy. Consequently, EM techniques that use electrons for imaging are the preferential methods to investigate at the nanoscale level.

**Figure 7:**
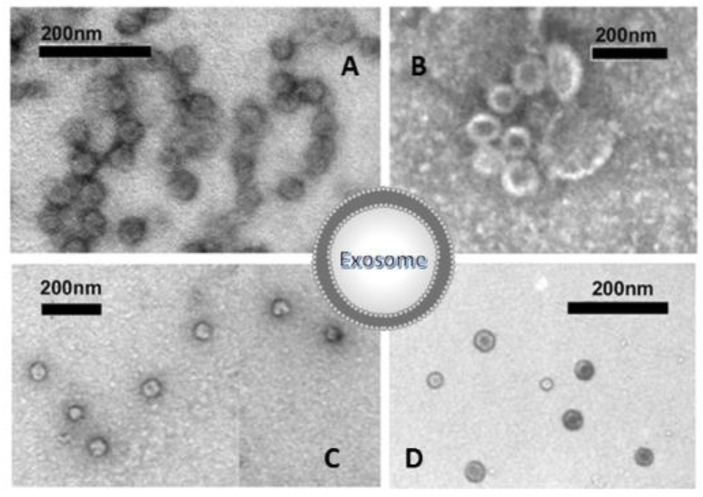
Electron microscopic view of individual exosomes. Whole-mount exosome preparations purified with ultracentrifugation and negative stained from plants (**A**), and different cell lines grown in culture (**B**–**D**). The cup-shaped morphology is clearly visible.

- *Imaging approaches* are critical steps in EXOs’ characterization; these usually include whole-mount SEM and TEM. The most accessible and simplest method of TEM imaging is conventional negative stain, which evidences a morphology called “cup-shaped”, because of a divot in the center of the exosomal vesicles (Figure 7). As reported by Raposo and Stoorvogel [9], the appearance of cup-shaped EXOs is likely an artifact due to the drying process associated with the sample preparation. Indeed, “whole-mount” samples are deposited on electron microscopy grids and allowed to desiccate on the surface. The nonuniformity of the capillary forces leads to a collapse resulting in a shape distortion. Accordingly, isolated frozen vesicles examined by cryo-EM and kept in their native state appear without ultrastructural changes; they show a spherical geometry rather than a cup-shaped feature. Moreover, 3D images generated by cryo-electron tomography and observations with other standard techniques verify the spherical shape.

In addition to spherical particles, EXOs can also appear oval or elongated [97,130]. An appropriate choice of grids, sample deposition, and TEM imaging conditions is essential for successful analysis [131].

**Figure 8:**
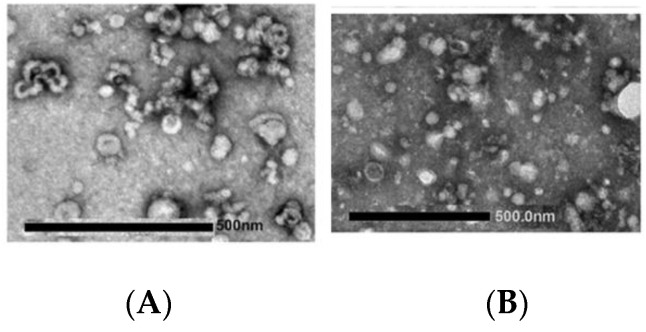
Transmission electron micrographs of exosome samples collected from cell culture and visualized after negative staining. In (**A**) the preparation is a population of large and small vesicles; the morphology is not uniform, some vesicles appear with a cup-like structure some with a deformed shape. Slight clumping can also be observed. In (**B**) vesicles are surrounded by microparticles, precipitates, and impurities generated in the stain or during preparation.

In addition to describing the morphology, EM can also provide an overview of the level of contamination of the sample confirming the sample purity; it can evidence the presence of larger vesicles, microparticles, apoptotic bodies, or cell debris such as protein aggregates, lipoproteins, and contaminant from cell cultures. Specific examples are shown in Figure 8.

The instructions given in MISEV2014 for EV characterization, and their updates in MISEV2018, suggest that EXOs must be analyzed using two different but complementary techniques, for example, electron and atomic force microscopy (AFM) [33].

AFM is a useful tool to investigate surface topology and material properties. Using a sharp tip mounted at the end of a micro-fabricated cantilever, AFM scans across the specimens immobilized on the support; AFM measures the force between the tip and the solid surface, thus allowing to assess the physical characteristics of single nanoparticles. Properties such as stiffness, adhesion, sample deformation or rigidity, and membrane fluidity are all of special significance for the transmembrane transport and biomedical function of vesicles. Compared with other high-resolution imaging and measurement techniques, AFM can simultaneously provide the structures and properties of biological samples under a desiccated form or under aqueous conditions, thus in their almost native states. However, EXO samples need to be immobilized on a mica substrate and then scanned: this can result in a change in the nanoparticle form, for example, by distorting their shape; in addition, when they are passively deposited from the liquid sample, the evaporation will produce drying artifacts [132,133].

- *Non-imaging methods,* such as dynamic light scattering (DLS), nanoparticle tracking analysis (NTA), Raman spectroscopy, tunable resistive pulse sensing (TRPS), and other biophysical approaches are being developed or adapted for EXOs data acquisition [134]. All these methods provide particles’ size distribution and concentration measurements; however, they indirectly estimate parameters via the use of some basic assumptions to interpret data. For example, NTA uses laser light to irradiate a nanoparticle suspension and estimates the size of EVs as their hydrodynamic diameters, which correlate the resistance to the Brownian mobility of EVs in the solution. A larger hydrodynamic diameter of a vesicle implies its lower mobility in liquid. Surface proteins and other molecules anchored or adsorbed to the membrane surface, the so-called the “coronal layer” around vesicles, and some debris can substantially influence the mobility and increases the hydrodynamic size of vesicles. Thus, their Brownian motion becomes difficult to track. DLS can quickly measure the average hydrodynamic diameter from a small volume of samples. An inherent weakness of DLS is its low resolution. To obtain satisfactory peak resolution, a particle size difference of at least three times is generally required. All these techniques are individually unable to determine the phenotype of the vesicles and their biochemical composition, and most commonly they need to be complemented with microscopy techniques.

For cytochemical/physical characterizations, new generation nano-flow cytometry methods, having detection limits as low as 100–200 nm, fluorescence (FM), and confocal laser scanning microscopy (CFM) are routinely used. These methods provide quantitative and qualitative information about EXOs. Indeed, if the vesicle bears some antigens at its surface, it is possible to detect them by applying fluorochrome-conjugated antibodies [135,136,137]. To obtain qualitative data on the chemical composition, specific histochemical techniques can be combined with the standard protocols; for example, specifically labeled markers can be bound in order to confirm the presence of proteins involved in MVB biogenesis.

Western blotting is the most commonly used technique to assess the presence of target proteins known to be associated with EVs. The principles behind Western blotting involve the specific binding of an antigen (target proteins) and an antibody that specifically recognizes the antigen. Western blot does not analyze intact vesicles; rather, sample preparation involves the lysis of the purified vesicles using buffered lysis solutions that contain denaturants and protease inhibitors. After denaturation, the protein lysates are separated by sodium dodecyl sulfate-polyacrylamide gel electrophoresis (SDS-PAGE), before being transferred over to a membrane for immunoblotting of specific protein. As suggested by guidelines in MISEV2018 [33], when WS is performed on EXOs from cell culture-conditioned medium, often a comparison with the material lysates from their producing cells needs to be performed to determine if proteins are associated with EXOs. WB analysis helps also to assess the degree of purity of the EV preparation. Indeed, for a WB analysis, EV-positive (proteins present or enriched in EVs/EXOs, such as CD63, CD81) and EV-negative (proteins not expected in EVs/EXOs such as calnexin and ribosomal protein S6 or others present in mitochondria, Golgi, or nuclear proteins) markers can be usually used. As such proteins may be present in larger or very large EVs, a single negative control may exclude the presence of such large EVs. Moreover, WB can evidence contaminants that co-isolate with EXOs (in biofluids such as blood plasma, proteins or lipoproteins have been reported to co-isolate); the guidelines in MISEV2018 [33] report the category of proteins to be considered when claiming specific analysis of small EVs. For biofluids, negative controls of disease-associated functions, for example, fluids from healthy, untreated, or otherwise matched donors, are performed.

The WB methodology is widely used for the analysis of EXOs due to its ease of use, wide accessibility, and ability to detect exosomal surface proteins and internal proteins. Nevertheless, the primary pitfall is its limited specificity due to the quality of several antibodies used and its lack of multiplicity, which results in the use of a large amount of exosomal protein employed to gain a minimal amount of information [138]. While the approach has a significant preparatory and processing time (>10 h), WB can provide useful information on the size of different proteins.

**Figure 9:**
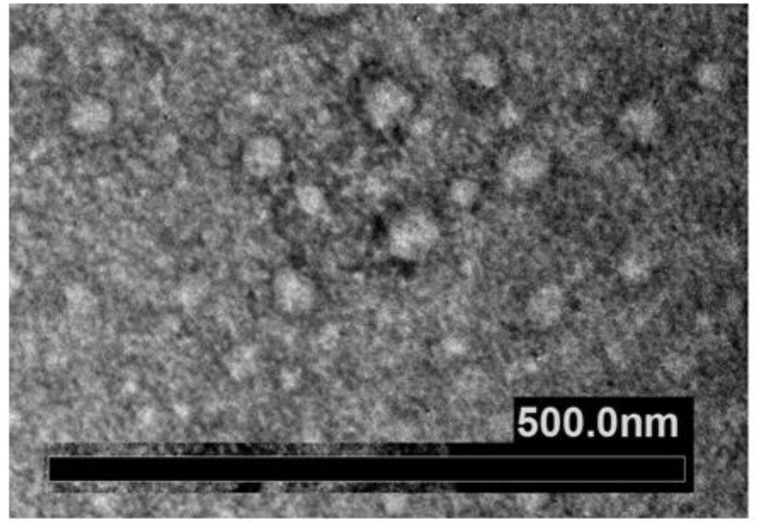
Immuno-electron detection and negative staining used to reveal the presence of exosome membrane protein labeled with gold particles.

For further characterization, immune-detection methods performed on TEM samples can be used to evidence specific molecules through their binding with colloidal gold-conjugated antibodies [130,139] (Figure 9).

To elucidate the uptake efficacy of nano-vesicles, the most common imaging techniques applied are conventional fluorescence microscopy (FM) and confocal fluorescence microscopy (CFM). They allow localizing fluorescently labeled EXOs (proteins or RNAs labeled) at the surface or inside target cells. Moreover, the combination of CFM with other imaging techniques such as flow cytometry and spectrofluorometric analysis enables obtaining information not only about internalization but also on the effect of functionalization in cell targeting and drug delivery. However, FM and flow cytometry methods lack detection accuracy and specificity as the resolution is insufficient to detect single nanovesicles or observe the interaction of vesicles with the cellular or tissue components.

The imaging and investigation of cellular fate and performance of EXOs often need high-resolving electron microscopy. SEM is a popular approach for acquiring information on their size, shape, and 3D surface topology; it provides information about the interaction of the vesicles with the plasma membrane of the target cells. On the contrary, TEM produces images at a higher resolution than SEM, and it can also be useful to better visualize the behavior of natural or engineered EXOs entering the cell. TEM allows investigating EXOs’ intracellular dynamics, how these vesicles are released from cells and how they are taken up by target cells, where they accumulate in the target site, and their intracellular persistence. Presently, TEM can provide details on the interaction of vesicles not only with the plasma membrane but also with the cytoplasmic organelles or the nuclear/sub-nuclear localization when entering the cell nucleus [140].

To avoid artifacts that can be introduced in the conventional preparation, or to reduce irradiation damages caused by an electron beam, a viable alternative is the use of TEM and SEM at cryogenic temperatures (cryo-TEM and cryo-SEM imaging). With this artifact-free approach, samples are analyzed at very low temperatures without fixation and staining; thus preserved in a near-native state through rapid freezing and imaged under cryogenic conditions [141,142,143]. However, these techniques remain a challenge due to the cost of the instruments, the expertise required to perform the sample preparation, imaging, and data analysis, and are time consuming because the observations often evidence a small number of particles in the images.

The relatively young field of correlative microscopy approaches, such as light microscopy and TEM methods, can help to overcome some limitations by offering at the same time the general and dynamic view of light microscopy and the ultrastructural information provided by TEM sometimes in combination with other various analytical methods [18,144].

Electron microscopy allows describing the EXOs’ producer cell or tissue, and furthermore, evidence of the structural and molecular alterations induced by nanovesicles on target cells [145].

**Figure 10:**
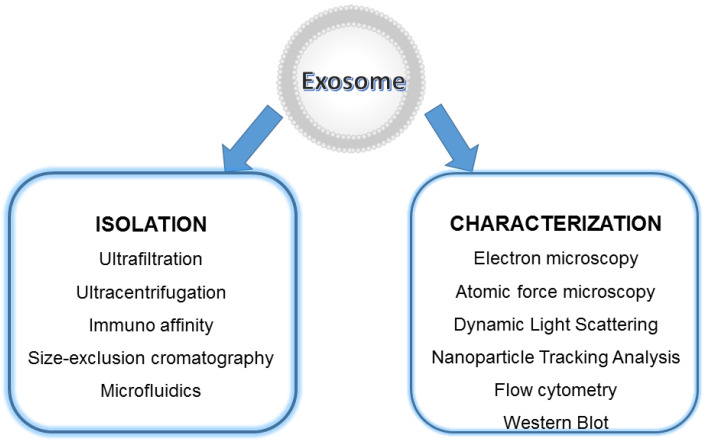
Isolation and characterization techniques for exosomal samples.

The identification of biomarkers for different diseases and cancers is often carried out via proteomic approaches used to identify the protein content or a predefined set of proteins within a sample. Thus, to control the quality of EVs isolation steps, and to characterize the abundance, ultrastructure, and biomolecular properties of EXOs, it is crucial to combine biochemical, microscopic investigations, and microanalytical techniques adapting classical and advanced methods of analysis (Figure 10).

## 6. Considerations and Challenges in the Research

Upon their initial discovery, EXOs were underestimated and were thought to function as waste systems. Over the following decades, very rapidly growing studies have instead proposed unique interesting functions of them.

EXOs are constitutively produced by all living cells including plants, animals, and microbes; they consist of membrane-enclosed structures containing a complex and well-coordinated mixture of biomolecules.

The major progress on EXOs was finding their active roles as new means of intercellular communication, both under healthy and pathological conditions. Nowadays, these nanoshuttles exerting a plethora of biological functions have aroused wide interest in biology and medical sciences and have been the subject of a very active field of research. They provide useful biomarkers for a variety of human diseases as a non-invasive diagnostic tool.

They are considered useful candidates to deliver compounds for therapeutics in various diseases. With this goal, EXOs can be manipulated to increase the dosage of drugs and to enhance tumor targeting; they can be loaded with a wide range of drugs, including chemotherapeutics compounds, DNA expression vectors, sRNA, and proteins. Furthermore, they have been shown to deliver their cargo, protecting it from degradation. Thus, drug delivery is one of their most promising applications in cancer therapy, neurological disorders, renal diseases, and many more. Several clinical trials are registered on the clinical trial database registry of the National Institute of Health (Home Clinical 2021).

However, before realizing the full potential of these therapeutic applications, a more comprehensive and detailed investigation of cytobiology needs to be performed. Safety evaluation is necessary in some contexts since the EVs may also show toxicity and immunogenicity that depend on many factors such as source, test model, or composition. Moreover, the different routes of administration such as intravenous, intraperitoneal, or subcutaneous injection, and nasal or oral administration, can also affect the biological distribution and the clearance of drugs and drug-loaded EXOs.

Many clinical trials on EXOs therapy have also been launched worldwide. Almost all EVs currently in phase I clinical trials and preclinical development are derived from cultured human cells (except for PureTech’s exosomes isolated from cow’s milk) and have been assessed in rodents or other animal models [146]. Thus, many questions are remaining to be answered:

- How are EXOs sorted to disparate targets?
- How can recipient cells discriminate among EVs? What are the mechanisms that mediate addressability?
- Which is the detailed mechanism of EXO uptake by recipient cells?
- The decision of EVs cargos’ destination in the recipient cells is not completely known. EXOs can release their cargo within the cytosol or directly within the nucleus or acidic organelles. Moreover, EXOs can also bind to extracellular matrix proteins regulating cell differentiation or promoting organ function repair. Fundamental questions remain about the fate of the biological cargo of EXOs.
- Do molecular cargos function singly or in combination with other EVs?
- Do EVs from different sources and distinct subtypes present different organ bio-distribution patterns and biological functions?
- Could the process of modification and loading with compounds of choice compromise EXOs biological functionality and induce immunogenicity?
- In addition, more data need to be collected on the characteristics of circulating EXOs and their spatiotemporal properties.

More systematic in vivo models combined with powerful imaging methods to track the biogenesis and fates of EVs will lead to a proper understanding of the basic functions and promote the translation of research into clinical applications. Moreover, in order to achieve sufficient quantities of these vesicles for clinical trials and to further improve their implementation for personalized medicines, especially in the field of oncology, it is essential to overcome main challenges and technical limitations such as:

- Inconsistent isolation methodologies and insufficient massive and stable production of engineered EXOs with constant characteristics for clinical use; methods currently available are time consuming and expensive;
- Lack of standardization of engineered EXO preparations, especially in a purified form, to ensure quality control;
- It is still not clear which cell source is mostly suitable for generation of engineered vesicles;
- Efficiency of cargo or inefficient loading in engineering EXOs: for example, it is currently lower than that for liposomes;
- Lack of selection methods of EVs subtypes as there is a high heterogeneity of subpopulations. The diverse subpopulations of EVs represent a major challenge for EVs-based theranostics applications. Apart from their heterogeneity in size, density, and shape that can be overcome by improving combinations of different separation methods, cell heterogeneity is an important question. The biogenesis of EXOs within each cell type heavily depends on the microenvironment as well as the physiological states, and EXOs are like a “fingerprint” of the releasing cell and its metabolic status. EVs cargo can be affected by changes in gene expression resulting from environmental cues such as oxygen levels or inflammation. Based on surface marker expression, Gebara et al. [147] found that amniotic fluid-derived EVs showed a heterogeneous origin; vesicles expressed markers of fetal, placental cells, and also mesenchymal and stem cells markers. Investigations on tetraspanin marker expression profiles in individual EVs have evidenced seven subpopulations. EXOs composition is not limited to proteins; lipids are other major components of EXOs, so the analysis of lipid expression in individual EVs may provide insight into their nature. Lipid biomolecules may affect target cell function via direct activation of cell surface receptors or as secondary messengers following endocytosis. Subpopulations may also be separated based on RNA profiles. Subpopulations of EXOs with different components could also originate from the different sorting machinery involved during their biogenesis. Indeed, EXOs formed from ESCRT-based pathways are associated with different envelope-distinct biomolecules compared to the EXOs that are formed independently from the ESCRT. Even when vesicles are isolated from a single cell source (e.g., from cells cultured in vitro), spatial and temporal changes in confluency, cell cycle stage, and stress may contribute to the observed heterogeneity. Circulating EXOs are released from a large variety of different cell types, and in a given disease, EVs from the relevant cell type or tissue may be masked or confounded by the contribution of cargo from multiple other cells. For such reason, which subpopulations of vesicles from blood provide information for disease diagnostics and which subpopulation is actually dictating the fate of the target cells remain unclear. To help overcome the heterogeneity challenges in EXOs-based applications, further investigations with a wide variety of additional EV markers are required. Various types of omics technologies to analyze at the single EXOs level need to be improved. Super-resolution microscopy is a promising approach that allows rapid and direct visualization of individual EVs and their surface protein markers in aqueous solution where the native structure is retained. Unfortunately, intra-EV visualization is extremely technically challenging due to the small and heterogeneous nature of EVs. A different new direction would be in vivo tracking and real-time imaging of subpopulations of EVs; studying the in vivo at a single vesicle level without disrupting their physiological environments is with a very high degree of complexity. The highly relevant question of whether EXOs subpopulations are truly functionally distinct remains to be answered. The complexity of EXOs requires a lot of effort to acquire the desired accuracy in isolation and separation procedures;
- Pharmaceutical parameters such as EV storage and stability are still not standardized. EVs obtained from different sources may require different storage conditions. Freeze/thaw cycles should be minimized, as they may damage the EV membranes. Adding cryoprotectants seems to be positive. To increase commercial availability, novel preservation methods are encouraged. Lyophilization seems to be a promising method for the preservation of vesicles, but its application requires further investigations, as studies reporting on this technique are still preliminary;
- Route of administration is an important factor affecting the safety of EXOs.

**Figure 11:**
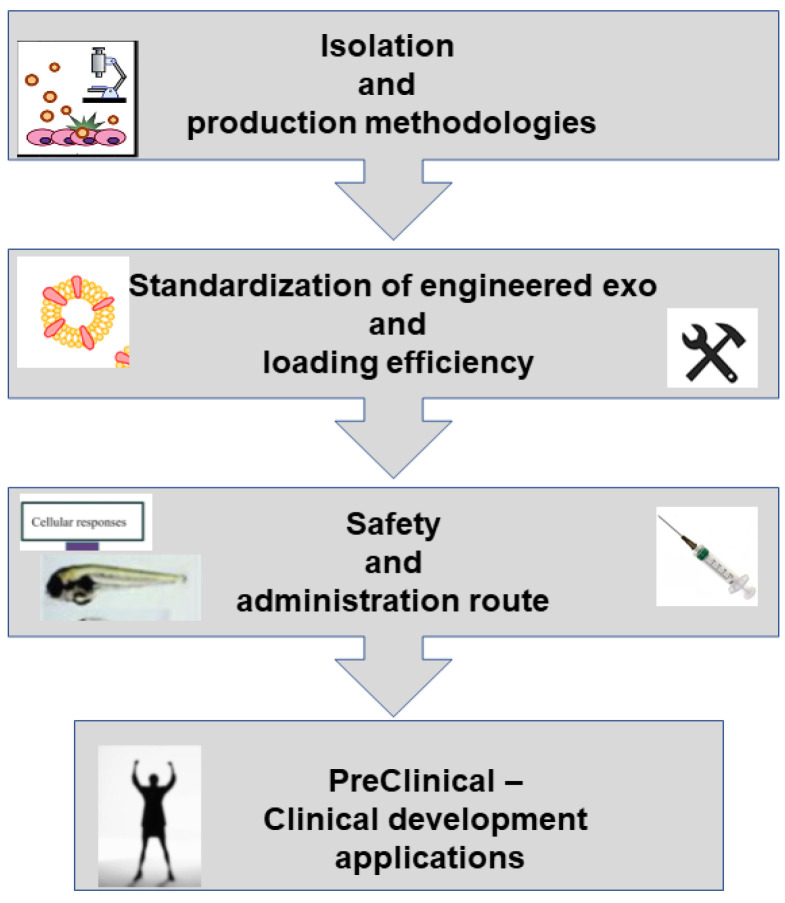
Different stages and challenges in the development of EV-based therapeutics. From the industrial-scale production of clinical-grade exosomes to the loading approaches and preservation of exosome biocompatibility, these bottlenecks need great efforts to accelerate the road to clinic.

Different stages that need a great deal of work to meet the standards for industrial and clinical application are summarized in Figure 11. For a detailed overview of these topics, the reader is referred to a recent review manuscript [148].

These limitations have stimulated the development of EXO nanotechnology in order to modify, engineer, and design them. Researchers have constructed and used biomimetic and bioinspired exosome-like nanovesicles as alternatives to natural ones, organic–inorganic hybridized, and synthetic nanoformulations called artificial EVs, the number of which is currently high. We direct the readers to the following recent reviews and position papers [25,83,149,150,151].

The exploration of the nanomedical applications of EXOs-based nano-platforms is an active area of research but is still in the early stages, although these nanoparticles represent a promising tool in the field of nanomedicine; at the same time, special regulatory guidelines in this area are urgently required. How can this be achieved soon? A lack of guidance in this field is a global issue; indeed, it is important to develop rigorous regulatory guidelines in nanomedicine [152].

## 7. Conclusions and Outlooks

Today, many studies are focused on improving the efficacy of traditional drugs and developing new therapies and new drug delivery systems for the targeting of diseased tissues. EXOs have received extensive attention from scientific communities. They have revealed high potential in the therapy of various diseases, especially in cancer, a disease that has a strong impact on human society; new innovative solutions to overcome the limitations derived from traditional chemotherapy and radiotherapy approaches will be developed using EXOs nano-carriers. In comparison to other synthetic loading drug systems, EXOs are more attractive and advantageous because of their natural origin. Although there are still many knowledge gaps to be filled, and significant challenges and difficulties in their application must be overcome, these endogenous vesicles show great potential to enter the clinic. This is an expanding research area, which involves many biological research areas and different fields of sciences; thanks to interdisciplinary approaches, it is expected that continued research and clinical efforts will evolve in the future providing innovation and optimizing the traditional therapeutic and diagnostic practice.

The primary goal of this review is to briefly summarize the main features of EVs and to address the recent advances in the field of EXO-based science with a focus on the isolation, characterization, and detection techniques useful for selecting and collecting them from cells. Aspects of their main applications in theranostics are given. Finally, the paper reports in short some considerations that are worthy of attention in order to better direct the efforts expected in the coming years for exosomal clinical transformation. This article has also the ambitious goal of contributing to orient, in a simple way, readers interested in this challenging area.

## Data Availability

Not applicable.

## References

[1] Drexler E.K., Peterson C., Pergamit G. (1991). Unbounding the Future: The Nanothechnology Revolution.

[2] Weissing V., Pettinger T.K., Murdock N. (2014). Nanopharmaceuticals (part 1): Products on the market. Int. J. Nanomed..

[3] Rizzo L.Y., Theek B., Storm G., Kiessling F., Lammers T. (2013). Recent progress in nanomedicine: Therapeutic, diagnostic and theranostic applications. Curr. Opin. Biotechnol..

[4] Kumar V., Bayda S., Hadla M., Caligiuri I., Russo Spena C., Palazzolo S., Kempter S., Corona G., Toffoli G., Rizzolio F. (2016). Enhanced Chemotherapeutic behavior of Open-caged DNA@Doxorubicin nanostructures for cancer cells. J. Cell Physiol..

[5] Chidambaran M., Manavalan R., Kathiresan K. (2011). Nanotherapeutics to overcome conventional cancer chemotherapy limitations. J. Pharm. Sci..

[6] Choi Y.H., Hang H. (2018). Nanomedicines: Current status and future perspectives in aspect of drug delivery and pharmacokinetics. J. Pharm. Investig..

[7] Wang J., Wu X., Shen P., Wang J., Shen Y., Shen Y., Webster T.J., Deng J. (2020). Applications of inorganic nanomaterials in pathothermal therapy based on combinational cancer treatment. Int. J. Nanomed..

[8] Brown L., Wolf J.M., Prados-Rosales R., Casadevall A. (2015). Through the wall: Extracellular vesicles in Gram-positive bacteria, mycobacteria and fungi. Nat. Rev. Microbiol..

[9] Raposo G., Stoorvogel W. (2013). Extracellular vesicles: Exosomes, microvesicles, and friends. J. Cell Biol..

[10] Colombo M., Raposo G., Thery C. (2014). Biogenesis, secretion, and intercellular interactions of exosomes and other extracellular vesicles. Annu. Rev. Cell Dev. Biol..

[11] Schwechheimer C., Kuehn M.J. (2015). Outer-membrane vesicles from Gram-negative bacteria: Biogenesis and functions. Nat. Rev. Microbiol..

[12] Valadi H., Ekström K., Bossios A., Sjöstrand M., Lee J.J., Lötvall J.O. (2007). Exosome- mediated transfer of mRNAs and microRNAs is a novel mechanisms of genetic exchange between cells. Nat. Cell Biol..

[13] Gangoda L., Boukouris S., Liem M., Kalra H., Mathivanan S. (2015). Extracellular vesicles including exosomes are mediators of signal transduction: Are they protective or pathogenic?. Proteomics.

[14] Tkach M., Théry C. (2016). Communication by extracellular vesicles: Where we are and where we need to go. Cell.

[15] Taylor D.D., Homesley H.D., Doellgast G.J. (1980). Binding of specific peroxidase-labe{}led antibody to placental-type phosphatase on tumor-derived membrane fragments. Cancer Res..

[16] Théry C., Zitvogel L., Amigorena S. (2002). Exosomes: Composition, biogenesis and function. Nat. Rev. Immunol..

[17] Lasser C., Alikhani V.S., Ekstrom K., Eldh M., Paredes P.T., Bossios A., Sjöstrand M., Gabrielsson S., Lötvall J., Valadi H. (2011). Human saliva, plasma and breast milk exosomes contain RNA: Uptake by macrophages. J. Transl. Med..

[18] Yuana Y., Koning R.I., Kuil M.E., Rensen P.C., Koster A., Bertina R.M., Osanto S. (2013). Cryo-electron microscopy of extracellular vesicles in fresh plasma. J. Extracell. Vesicles.

[19] Arraud N., Linares R., Tan S., Gounou C., Pasquet J.M., Mornet S., Brisson A.R. (2014). Extracellular vesicles from blood plasma: Determination of their morphology, size, phenotype and concentration. J. Thromb. Haemost..

[20] Greening D.W., Gopal S.K., Mathias R.A., Liu L., Sheng J., Zhu H.J., Simpson R.J. (2015). Emerging roles of exosomes during epithelial-mesenchymal transition and cancer progression. Semin. Cell Dev. Biol..

[21] Hoog J.L., Lotvall J. (2015). Diversity of extracellular vesicles in human ejaculates revealed by cryo-electron microscopy. J. Extracell. Vesicles.

[22] Van Dommelen S.M., Vader P., Lakhal S., Kooijmans S.A., van Solinge W.W., Wood M.J., Schiffelers R.M. (2012). Microvesicles and exosomes: Opportunities for cell-derived membrane vesicles in drug delivery. J. Control. Release.

[23] Tan A., Rajadas J., Seifalian A.M. (2013). Exosomes as nano-theranostic delivery platforms for gene therapy. Adv. Drug Deliv. Rev..

[24] Batrakova E.V., Kim M.S. (2015). Using exosomes, naturally-equipped nanocarriers, for drug delivery. J. Control. Release.

[25] Yang B., Chen Y., Shi J. (2019). Exosome biochemistry and advances nanotechnology for next–generation theranostic platform. Adv. Mater..

[26] Ha D., Yang N., Nadithe V. (2016). Exosomes as therapeutic drug carriers and delivery vehicles across biological membranes: Current perspectives and future challenges. Acta Pharm. Sin. B.

[27] Shi M., Sheng l., Stewart T., Zabetian C.P., Zhang J. (2019). New windows into the brain: Central nervous system-derived extracellular vesicles in blood. J. Prog. Neurobiol..

[28] Pan B.T., Teng K., Wu C., Adam M., Johnstone R.M. (1985). Electron microscopic evidence for externalization of the transferrin receptor in vesicular form in sheep reticulocytes. J. Cell Biol..

[29] Crescitelli R., Lasser C., Szabo T.G., Kittel A., Eldh M., Dianzani I., Buzás E.I., Lötvall J. (2013). Distinct RNA profiles in subpopulations of extracellular vesicles: Apoptotic bodies, microvesicles and exosomes. J. Extracell. Vesicles.

[30] Brydson R., Brown A., Hodges C., Abellan P., Hondow N. (2015). Microscopy of nanoparticulate dispersions. J. Microsc..

[31] Szatanek R., Baj-Krzyworzeka M., Zimoch J., Lekka M., Siedlar M., Baran J., Szatanek R., Baj-Krzyworzeka M., Zimoch J. (2017). The methods of choice for Extracellular Vesicles (EVs) Characterization. Int. J. Mol. Sci..

[32] Meldolesi J. (2018). Exosomes and ectosomes in intercellular communication. Curr. Biol..

[33] Théry C., Witwer K.W., Aikawa E., Alcaraz M.J., Anderson J.D., Andriantsitohaina R., Antoniou A., Arab T., Archer F., Atkin-Smith G.K. (2018). Minimal information for studies of extracellular vesicles 2018 (MISEV2018): A position statement of the international society for extracellular vesicles and update of the MISEV2014 guidelines. J. Extracell. Vesicles.

[34] Kerr J.F., Wyllie A.H., Currie A.R. (1972). Apoptosis: A basic biological phenomenon with wide ranging implications in tissue Kinetics. Br. J. Cancer.

[35] Elmore S. (2007). Apoptosis: A review of programmed cell death. Toxicol. Pathol..

[36] Poon I.K.H., Lucas C.D., Rossi A.G., Ravichandran K.S. (2014). Apoptotic cell clearance: Basic biology and therapeutic potential. Nat. Rev. Immunol..

[37] Xu X.B., Lai Y.Y., Zi C.H. (2019). Apoptosis and apoptotic body: Disease message and therapeutic target potentials. Biosci. Rep..

[38] Lee Y., El Andaloussi S., Wood M.J. (2012). Exosomes and microvesicles: Extracellular vesicles for genetic information transfer and gene therapy. Hum. Mol. Genet..

[39] Cocucci E., Meldolesi J. (2015). Ectosomes and exosomes: Shedding the confusion between extracellular vesicles. Trends Cell Biol..

[40] Kamerkar S., LeBleu V.S., Sugimoto H., Yang S., Ruivo C.F., Melo S.A., Lee J.J., Kalluri R. (2017). Exosomes facilitate therapeutic targeting of oncogenic KRAS in pancreatic cancer. Nature.

[41] Elsharkasy O.M., Nordin J.Z., Hagey D.W., de Jong O.G., Schiffelers R.M., Andaloussi S.E.L., Vader P. (2020). Extracellular vesicles as drug delivery systems: Why and how?. Adv. Drug. Deliv. Rev..

[42] Wolf P. (1967). The nature and significance of platelet products in human plasma. Br. J. Haematol..

[43] Pan B.T., Johnstone R.M. (1983). Fate of the tranferrin receptor during maturation of sheep reticulocytes in vitro: Selective externalization of the receptor. Cell.

[44] Harding C., Sthahl P. (1983). Tranferrin recycling in reticulocytes: pH and iron are important determinants of ligand binding and processing. Biochem. Biophys. Res. Commun..

[45] Johnstone R.M., Adam M., Hammond J.R., Orr L., Turbide C. (1987). Vesicle formation during reticulocyte maturation. Association of plasma membrane activities with released vesicles (exosomes). J. Biol. Chem..

[46] Théry C. (2011). Exosomes: Secreted vesicles and intercellular communications. F1000 Biol. Rep..

[47] Rashed M.H., Bayraktar E., Helal G.K., Abd-Ellah M.F., Amero P., Chavez-Reyes A., Rodriguez-Aguayo C. (2017). Exosomes: From garbage bins to promising therapeutic targets. Int. J. Mol. Sci..

[48] Van Niel G., Porto-Carreiro I., Simoes S., Raposo G. (2006). Exosomes: A common pathway for a specialized function. J. Biochem..

[49] Lotwall J., Hill A.F., Hochberg F., Buzàs E.J., Di Vizio D., Gardiner C., Gho Y.S., Kurochkin I.V., Mathivanan S., Quesenberry P. (2014). Minimal experimental requirements for definition of extracellular vesicles and their functions: A position statement from the International Society for Extracellular Vesicles. J. Extracell. Vesicles.

[50] Kowal J., Arras G., Colombo M., Jouve M., Morat J.P., Primdal-Bengtson B., Dingli F., Loew D., Tkach M., Thery C. (2016). Proteomic comparison defines novel markers to characterize heterogeneous populations of extracellular vesicles subtypes. Proc. Natl. Acad. Sci. USA.

[51] Kalluri R., le Bleu V.S. (2020). The biology, function, and biomedical applications of exosomes. Science.

[52] Jeppesen D., Fenix A.M., Franklin J.L., Higginbotham J.N., Zhang Q., Zimmerman L.J., Liebler D.C., Ping J., Liu Q., Evans R. (2019). Reassessment of exosome composition. Cell.

[53] Mulkahy L.A., Pink R.C., Carter D.R.F. (2014). Routes and mechanisms of extracellular vesicle uptake. J. Extracell. Vesicles.

[54] Van Niel G., d’Angelo G., Raposo G. (2018). Shedding light on the cell biology of extracellular vesicles. Nat. Rev. Mol. Cell Biol..

[55] Mathieu M., Martin-Jaular L., Lavieu G., Thery C. (2019). Specificities of secretion and uptake of exosomes and other extracellular vesicles for cell-to-cell communication. Nat. Cell Biol..

[56] Record M., Carayon K., Poirot M., Silvente-Poirot S. (2014). Exosomes as new vesicular lipid transporters involved in cell-cell communication and various pathophysiologies. Biochim. Biophys. Acta.

[57] Zhou C.F., Fong M.Y., Min Y., Somlo G., Liu L., Palomares M.R., Yu Y., Chow A., O’Connor S.T., Chin A.R. (2014). Cancer secreted miR-105 destroys vascular endothelium barriers to promote metastasis. Cancer Cell.

[58] Lafourcade C., Ramirez J.P., Luarte A., Fernandez A., Wyneken U. (2016). MiRNA in astrocyte-derived-exosomes as possible mediators of neuronal plasticity. J. Exp. Neurosci..

[59] Herrera M., Llorens C., Rodriguez M., Herrera A., Ramos R., Gil B., Candia A., Larriba M.J., Garre P., Earl J. (2018). Differential distribution and enrichment of non-coding RNAs in exosomes from normal and cancer-associated fibroblasts in colorectal cancer. Mol. Cancer.

[60] Chen Z., Larregina A.T., Morelli A.E. (2019). Impact of extracellular vesicles on innate immunity. Curr. Opin. Organ Transplant..

[61] Zhou C.F., Ma J., Huang L., Yi H.Y., Zhang Y.M., Wu X.G., Yan R.M., Liang L., Zhong M., Yu Y.H. (2019). Cervical squamous cell carcinoma-secreted exosomal miR-221-3p promotes lymphangiogenesis and lymphatic metastasis by targeting VASH1. Oncogene.

[62] Rezaie J., Rahbarghazi R., Pezeshki M., Mazhar M., Yekani F., Khaksar M., Shokrollahi E.H., Hashemzadeh S., Sokullu S., Tokac M. (2019). Cardioprotective role of extracellular vesicles: A highlight on exosome beneficial effects in cardio-vascular diseases. J. Cell Physiol..

[63] Li L., Li C., Wang S., Wang Z., Jiang J., Wang W., Li X., Chen J., Liu K., Li C. (2016). Exosomes derived from hypoxic oral squamous cell carcinoma cells deliver miR-21 to normoxic cells to elicit a prometastatic phenotype. Cancer Res..

[64] Taverna S., Pucci M., Giallombardo M., Di Bella M.A., Santarpia M., Reclusa P., Bazo I.B., Rolfo C., Alessandro R. (2017). Amphiregulin contained in NSCLC exosomes induces osteoclast differentiation through the activation of EGFR pathway. Sci. Rep..

[65] Rajagopal C., Harikumar K.B. (2018). The origin and functions of exosomes in cancer. Front. Oncol..

[66] Li L.M., Liu Z.X., Cheng Q.Y. (2019). Exosome plays an important role in the development of hepatocellular carcinoma. Pathol. Res. Pract..

[67] Rahbarghazi R., Jabbari N., Sani N.A. (2019). Tumor derived extracellular vesicles: Reliable tools for cancer diagnosis and clinical applications. Cell Commun. Signal.

[68] Meng W., He C., Hao Y., Wang L., Li L., Zhu G. (2020). Prospects and challenges of extracellular vesicle-based drug delivery system: Considering cell source. Drug Deliv..

[69] Pitt J.M., André F., Amirogena S., Soria J.C., Eggermont A., Kroemer G., Zitvogel L. (2016). Dendritic cell-derived exosomes for cancer therapy. J. Clin. Investig..

[70] Liu Q., Rojas-Canales D.M., Divito S.J., Shufesky W.J., Stolz D.B., Erdos G., Sullivan M.L., Gibson G.A., Watkins S.C., Larregina A.T. (2016). Donor dendritic cell-derived exosomes promote allograft-targeting immune response. J. Clin. Investig..

[71] Khan A.R., Yang X., Fu M., Zhai G.J. (2018). Recent progress of drug nano-formulations targeting to brain. J. Control. Release.

[72] Pullan J.E., Confeld M.I., Osborn J.K., Kim J., Sarkar K., Mallik S. (2019). Exosomes as drug carriers for cancer therapy. Mol. Pharm..

[73] Cheng L., Wang Y., Huang L. (2017). Exosomes from M1-Polarized Macrophages Potentiate the Cancer Vaccine by Creating a Pro-inflammatory Microenvironment in the Lymph Node. Mol. Ther..

[74] Yuan Z.H., Petree J.R., Lee F.E.H., Fan X., Salaita K., Guidot D.M., Sadikot R.T. (2019). Macrophages exposed to HIV viral protein disrupt lung epithelial cell integrity and mitochondrial bioenergetics via exosomal microRNA shuttling. Cell Death Dis..

[75] Cao J., Wang B., Tang T., Wen Y., Li Z., Feng S., Wu M., Liu D., Yin D., Ma K. (2021). Exosomal miR-125b-5p deriving from mesenchymal stem cells promotes tubular repair by suppression of p53 in ischemic acute kidney injury. Theranostics.

[76] Zhang B., Yin Y., Lai R.C., Tan S.S., Choo A.B.H., Lim S.K. (2014). Mesenchymal stem cell secrete immunologically active exosomes. Stem Cells Dev..

[77] Gégroire V., Langendijk J.A. (2015). Advances in radiotherapy for head and neck cancer. J. Clin. Oncol..

[78] Yao K., Ricardo S.D. (2016). Mesenchymal stem cell as novel micro-ribonucleic acid delivery vehicles in kidney disease. Nephrology.

[79] Che Y., Shi X., Shi Y., Jiang X., Ai Q., Shi Y., Gong F., Jiang W. (2019). Exosomes derived from miR-143-oerexpressing MSCs inhibit cell migration and invasion in human prostate cancer by downregulating TFF3. Mol. Ther. Nucleic Acids.

[80] Qu L., Ding J., Chen C., Wu Z.J., Liu B., Gao Y., Chen W., Liu F., Sun W., Li X.F. (2016). Exosome-transmitted lncARSR promotes Sunitinib resistance in renal cancer by acting as a competing endogenous RNA. Cancer Cell..

[81] Qiao L., Hu S., Huang K., Su T., Li Z., Vandergriff A., Cores J., Dinh P.U., Allen T., Shen D. (2020). Tumour cell-derived exosomes home to their cells of origin and can be used as Trojan horses to deliver cancer drugs. Theranostics.

[82] Usman W.M., Pham T.C., Kwok Y.Y. (2018). Efficient RNA drug delivery using red blood cells extracellular vesicles. Nat. Commun..

[83] Zhang X., Zhang H., Gu J., Zhang J., Shi H., Quan H., Wang D., Xu W., Pan J., Santos H.A. (2021). Engineered Extracellular vesicles for cancer therapy. Adv. Mater..

[84] Melnik B.C., John S.M., Schmitz G. (2014). Milk: An exosomal microRNA transmitter promoting thymic regulatory T cell maturation preventing the development of atopy?. J. Transl. Med..

[85] Agrawal A.K., Aqil F., Jeyabalan J., Spencer W.A., Beck J., Gachuki B.W., Alhakeem S.S., Oben K., Munagala R., Bondada S. (2017). Milk-derived exosomes for oral delivery of paclitaxel. Nanomedicine.

[86] Wang Q., Zhuang X., Mu J., Deng Z.B., Jiang H., Zhang L., Xiang X., Wang B., Yan J., Miller D. (2013). Delivery of the therapeutic agents by nanoparticles made of grapefruit-derived lipid. Nat. Commun..

[87] Zhang M., Viennois E., Prasad M., Zhang Y., Wang L., Zhang Z., Han M.K., Xiao B., Xu C., Srinivasan S. (2016). Edible ginger-derived nanoparticles: A novel therapeutic approach for the prevention and treatment of inflammatory bowel disease and colitis-associated cancer. Biomaterials.

[88] Di Gioia S., Hossain N., Conese M. (2020). Biological properties and therapeutic effects of plant-derived nanovesicles. Open Med..

[89] Yang C., Zhang M., Merlin D.A. (2018). Advances in plant-derived edible nanoparticle-based lipid nano-drug delivery systems as therapeutic nanomedicines. J. Mater. Chem. B.

[90] Raimondo S., Naselli F., Fontana S., Monteleone F., Lo Dico A., Saieva L., Zito G., Flugy A., Manno M., Di Bella M.A. (2015). Citrus Limon-derived nanovesicles inhibit cancer cell proliferation and suppress CML xenograft growth by inducing TRAIL –mediated cell death. Oncotarget.

[91] Cheng Y., Zeng Q., Han Q., Xia W. (2019). Effect of pH, temperature and freezing-thawing on quantity changes and cellular uptake of exosomes. Protein Cell.

[92] Xu R., Simpson R.J., Greening D.W.A. (2017). Protocol for isolation and proteomic characterization of distinct extracellular vesicle subtypes by sequential centrifugal ultrafiltration. Methods Mol. Biol..

[93] Tauro B.J., Greening D.W., Mathias R.A., Ji H., Mathivanan S., Scott A.M., Simpson R.J. (2012). Comparison of ultracentrifugation, density gradient separation, and immunoaffinity capture methods for isolating human colon cancer cell line LIM1863-derived exosomes. Methods.

[94] Boing A.N., van der Pol E., Grootemaat A.E., Coumans F.A., Sturk A., Nieuwland R. (2014). Single step isolation of extracellular vesicles by size- exclusion chromatography. J. Extracell. Vesicles.

[95] Zhang H., Freitas D., Kim H.S., Fabijanic K., Li Z., Chen H., Mark M.T., Molina H., Martin A.B., Bojmar L. (2018). Identification of distinct nanoparticles and subsets of extracellular vesicles by asymmetric flow field-flow fractionation. Nat. Cell Biol..

[96] Liangsupree T., Multia E., Riekkola M.L. (2021). Modern isolation and separation techniques for extracellular vesicles. J. Chromatogr. A.

[97] Théry C., Amigorena S., Raposo G., Clayton A. (2006). Isolation and characterization of exosomes from cell culture supernatants and biological fluids. Curr. Protoc. Cell Biol..

[98] Rider M.A., Hurwitz S.N., Meckes D.G. (2016). ExtraPEG: A polyethylene glycol-based method for enrichment of extracellular vesicles. Sci. Rep..

[99] Chen J., Xu Y., Lu W., Xing W. (2018). Isolation and visible detection of tumor-derived exosomes from plasma. Anal. Chem..

[100] Wu J.Y., Li Y.J., Hu X.B., Huang S., Xian D.X. (2021). Preservation of small extracellular vesicles for functional analysis and therapeutic applications: A comparative evaluation of storage conditions. Drug Deliv..

[101] Ke D., Li H., Zhang Y., An Y., Fu H., Fang X., Zheng X. (2017). The combination of circulating long noncoding RNAs AK001058, INHBA-AS1, MIR4435-2HG, and CEBPA-AS1 fragments in plasma serve as diagnostic markers for gastric cancer. Oncotarget.

[102] Kosaka N., Kogure A., Yamamoto T., Urabe F., Usuba W., Prieto-Vila M., Ochiya T. (2019). Exploiting the message from cancer: The diagnostic value of extracellular vesicles for clinical applications. Exp. Mol. Med..

[103] Shaimardanova A.A., Solovyeva V.V., Chulpanova D.S., James V., Kitaeva K.V., Rizvanov A.A. (2020). Extracellular vesicles in the diagnosis and treatment of central nervous system diseases. Neural. Regen. Res..

[104] Sandfeld-Paulsen N., Aggerholm-Pedersen R., Bæk K.R., Jakobsen P., Meldgaard P., Folkersen B.H., Rasmussen T.R., Varming K., Jørgensen M.M., Sorensen B.S. (2016). Exosomal proteins as prognostic biomarkers in non-small cell lung cancer. Mol. Oncol..

[105] Grimolizzi F., Monaco F., Leoni F., Bracci M., Staffolani S., Bersaglieri C., Gaetani S., Valentino M., Amati M., Rubini C. (2017). Exosomal miR-126 as a circulating biomarker in non-small-cell lung cancer regulating cancer progression. Sci. Rep..

[106] Porzycki P., Ciszkowicz E., Semik M., Tyrka M. (2018). The combination of three miRNA (miR-141, miR-21, and miR-375) as potential diagnostic tool for prostate cancer recognition. Int. Urol. Nephrol..

[107] Fiandaca M.S., Kapogiannis D., Mapstone M., Boxer A., Eitan E., Schwartz J.B., Abner E.L., Petersen R.C., Federoff H.J., Miller B.L. (2015). Identification of preclinical Alzheimer’s disease by a profile of pathogenic proteins in neurally derived blood exosomes: A case-control study. Alzheimer’s Dement..

[108] Krause M., Samoylenko A., Vainio S.J. (2015). Exosomes as renal inductive signals in health and disease, and their application as diagnostic markers and therapeutic agents. Front. Cell Dev. Biol..

[109] Dorronsoro A., Robbins P.D. (2013). Regenerating the injured kidney with human umbilical cord mesenchymal stem cell-derived exosomes. Stem Cell Res. Ther..

[110] Long Q., Upadhya D., Hattiangady B., Kim D.K., An S.Y., Shuai B., Prockop D.J., Shetty A.K. (2017). Intranasal MSC-derived A1-exosomes ease inflammation, and prevent abnormal neurogenesis and memory dysfunction after status epilepticus. Proc. Natl. Acad. Sci. USA.

[111] Otero-Ortega L., Laso-García F., Gómez-de Frutos M.D., Rodríguez-Frutos B., Pascual-Guerra J., Fuentes B., Díez-Tejedor E., Gutiérrez-Fernández M. (2017). White Matter repair after Extracellular Vesicles administration in an experimental animal model of subcortical Stroke. Sci. Rep..

[112] Cantaluppi V., Gatti S., Medica D., Figliolini F., Bruno S., Deregibus M.C., Sordi A., Biancone L., Tetta C., Camussi G. (2012). Microvesicles derived from endothelial progenitor cells protect the kidney from ischemia-reperfusion injury by microRNA-dependent reprogramming of resident renal cells. Kidney Int..

[113] Cosenza S., Ruiz M., Toupet K., Jorgensen C., Noël D. (2017). Mesenchymal stem cells derived exosomes and microparticles protect cartilage and bone from degradation in osteoarthritis. Sci. Rep..

[114] De Carvalho J.V., De Castro R.O., da Silva E.Z.M., Silveira P.P., Da Silva-Januário M.E., Arruda E., Jamur M.C., Oliver C., Aguiar R.S., Dasilva L.L.P. (2014). Nef neutralizes the ability of exosomes from CD4^+^ T Cells to Act as decoys during HIV-1 infection. PLoS ONE.

[115] Keller M.D., Ching K.L., Liang F.X., Dhabaria A., Tam K., Ueberheide B.M., Unutmaz D., Torres V.J., Cadwell K. (2020). Decoy exosomes provide protection against bacterial toxins. Nature.

[116] Cocozza F., Névo N., Piovesana E., Lahaye X., Buchrieser J., Schwartz O., Manel N., Tkach M., Théry C., Martin-Jaular L. (2020). Extracellular vesicles containing ACE efficiently prevent infection by SARS-CoV-2 Spike protein-containing virus. J. Extracell. Vesicles.

[117] Inal J.M. (2020). Decoy ACE2-expressing extracellular vesicles that competitively bind SARS-CoV-2 as a possible COVID-19 therapy. Clin. Sci..

[118] Kumar S., Zhi K., Mukherji A., Gerth K. (2020). Repurposing antiviral protease inhibitors using extracellular vesicles for potential therapy of COVID-19. Viruses.

[119] Hassanpour M., Rezaie J., Nouri M., Panahi Y. (2020). The role of extracellular vesicles in COVID-19 virus infection. Infect. Genet. Evol..

[120] Troyer Z., Alhusaini N., Tabler C.O., Sweet T., de Carvalho K.I.L., Schlatzer D.M., Carias L., King C.L., Matreyek K., Tilton J.C. (2021). Extracellular vesicles carry SARS-CoV-2 spike protein and serve as decoys for neutralizing antibodies. J. Extracell. Vesicles.

[121] Yoo K.H., Thapa N., Kim B.J., Lee J.O., Jang Y.N., Chwae Y.J., Kim J. (2022). Possibility of exosome-based coronavirus disease 2019 vaccine. Mol. Med. Rep..

[122] Sun D., Zhuang X., Xiang X., Liu Y., Zhang S., Liu C., Barnes S., Grizzle W., Miller D., Zhang H.G. (2010). A novel nanoparticle drug delivery system: The anti-inflammatory activity of curcumin is enhanced when encapsulated in exosomes. Mol. Ther..

[123] Yang T., Martin P., Fogarty B., Brown A., Schurman K., Phipps R., Yin V.P., Lockman P., Bai S. (2015). Exosome delivered anticancer drugs across the blood-brain barrier for brain cancer therapy in Danio rerio. Pharm. Res..

[124] Balachandran B., Yuana Y., Schumacher U. (2019). Extracellular vesicles-based drug delivery system for cancer treatment. Cogent Med..

[125] Fu S., Wang Y., Xia X., Zheng J.C. (2020). Exosome engineering: Current progress in cargo loading and targeted delivery. NanoImpact.

[126] Smyth T., Kullberg M., Malik N., Smith-Jones P., Graner M.W., Anchordoquy T.J.J. (2015). Biodistribution and delivery efficiency of unmodified tumor-derived exosomes. J. Control. Release.

[127] Wang C., Wang A., Wei F., Wong D.T.W., Tu M. (2017). Electric field-induced disruption and realizing viable content from extracellular vesicles. Methods Mol. Biol..

[128] Joshi B.S., de Beer M.A., Giepmans B.N.G., Zuhorn I.S. (2020). Endocytosis of extracellular vesicles and release of their cargo from endosomes. ACS Nano.

[129] Hettich B.F., Bader J.J., Leroux J.C. (2021). Encapsulation of hydrophilic compounds in small extracellular vesicles: Loading capacity and impact on vesicle functions. Adv. Healthc. Mater..

[130] Zabeo D., Cvjetkovic A., Lasser C., Schorb M., Lotvall J., Hoog J.L. (2017). Exosomes purified from a single cell type have diverse morphology. J. Extracell. Vesicles.

[131] Rikkert L.G., Nieuwland R., Terstappen L.W.M.M., Coumans F.A.W. (2019). Quality of extracellular vesicle images by transmission electron microscopy is operator and protocol dependent. J. Extracell. Vesicles.

[132] Sharma S., LeClaire M., Gimzewski J.K. (2018). Ascent of atomic force microscopy as a nanoanalytical tool for exosomes and other extracellular vesicles. Nanotechnology.

[133] Skliar M., Chernyshev V.S. (2019). Imaging of extracellular vesicles by atomic force microscopy. J. Vis. Exp..

[134] Dragovic R.A., Gardiner C., Brooks A.S., Tannetta D.S., Ferguson D.J.P., Hole P., Carr B., Redman C.W.G., Harris A.L., Dobson P.J. (2017). Sizing and phenotyping of cellular vesicles using nanoparticle tracking analysis. Nanomed. Nanotechnol. Biol. Med..

[135] Orozco A.F., Lewis D.E. (2010). Flow cytometric analysis of circulating microparticles in plasma. Cytometry A.

[136] Shen W., Guo K., Adkins G.B., Jiang Q., Liu Y., Sedano S., Duan Y., Yan S., Wang E., Bergersen K. (2018). A single extracellular vesicle (EV) flow cytometry approach to reveal EV heterogeneity. Angew. Chem..

[137] Choi D., Montermini L., Jeong H., Sharma S., Meehan B., Rak J. (2019). Mapping subpopulations of cancer cell-derived extracellular vesicles and particles by nano-flow cytometry. ACS Nano.

[138] Doyle L.M., Wang M.Z. (2019). Overview of extracellular vesicles, their origin, composition, purpose, and methods for exosomes isolation and analysis. Cells.

[139] Semina S.E., Scherbakov A.M., Vnukova A.A., Bagrov D.V., Evtushenko E.G., Safronova V.M., Golovina D.A., Lyubchenko L.N., Gudkova M.V., Krasil’nikov M.A. (2018). Exosome-mediated transfer of cancer cell resistance to antiestrogen drugs. Molecules.

[140] Rappa G., Santos M.F., Green T.M., Karbanova J., Hassler J., Bai Y., Barsky S.H., Corbeil D., Lorico A. (2017). Nuclear transport of cancer extracellular vesicle-derived biomaterials through nuclear envelope invagination-binding protein-associated late endosomes. Oncotarget.

[141] Koifman N., Biran I., Aharon A., Brenner B., Talmon Y.A. (2017). Direct-imaging cryo-EM study of shedding extracellular vesicles from leukemic monocytes. J. Struct. Biol..

[142] Cizmar P., Yuana Y. (2017). Detection and characterization of extracellular vesicles by transmission and cryo-transmission electron microscopy. Methods Mol. Biol..

[143] Noble J.M., Roberts L.D., Vidavsky N., Chiou A.E., Fischbach C., Paszeka M.J., Estroffc L., Kourkoutis L.F. (2020). Direct comparison of optical and electron microscopy methods for structural characterization of extracellular vesicles. J. Struct. Biol..

[144] Polishchuk E.V., Polishchuk R.S., Luini A. (2013). Correlative light-electron microscopy as a tool to study in vivo dynamics and ultrastructure of intracellular structures. Methods Mol. Biol..

[145] Schillaci O., Fontana S., Monteleone F., Taverna S., Di Bella M.A., Di Vizio D., Alessandro R. (2017). Exosomes from metastatic cancer cells transfer amoeboid phenotype to non-metastatic cells and increase endothelial permeability: Their emerging role in tumor heterogeneity. Sci. Rep..

[146] Cully M. (2021). Exosome-based candidates move into the clinic. Nat. Rev. Drug Discov..

[147] Gebara N., Scheel J., Skovronova R., Grange C., Marozio L., Gupta S., Giorgione V., Caicci F., Benedetto C., Khalil A. (2022). Single extracellular vesicle analysis in human amniotic fluid shows evidence of phenotype alterations in preeclampsia. J. Extracell. Vesicles.

[148] Murphy D.E., de Jong O.G., Brouwer M., Wood M.J., Lavieu G., Schiffelers R.M., Vader P. (2019). Extracellular vesicle-based therapeutics: Natural versus engineered targeting and trafficking. Exp. Mol. Med..

[149] Garcia-Manrique P., Matos M., Gutierrez G., Pazos C., Blanco-Lopez M.C. (2018). Therapeutic biomaterials based on extracellular vesicles: Classification of bio-engineering and mimetic preparation routes. J. Extracell. Vesicles.

[150] Wu P., Zhang B., Ocansey D.K.W., Xu W., Qian H. (2021). Extracellular vesicles: A bright star of nanomedicine. Biomaterials.

[151] Chan M.H., Chang Z.X., Huang C.F., Lee L.J., Liu R.S., Hsiao M. (2022). Integrated therapy platform of exosomal system: Hybrid inorganic/organic nanoparticles with exosomes for cancer treatment. Nanoscale Horiz..

[152] Foulkers R., Man E., Thind J., Yeung S., Joy A., Hoskins C. (2020). The regulation of nanomaterials and nanomedicine for clinical application: Current and future perspectives. Biomater. Sci..

